# Overexpression of FoxM1 optimizes the therapeutic effect of bone marrow mesenchymal stem cells on acute respiratory distress syndrome

**DOI:** 10.1186/s13287-023-03240-8

**Published:** 2023-02-14

**Authors:** Yuling Luo, Shanhui Ge, Qingui Chen, Shan Lin, Wanmei He, Mian Zeng

**Affiliations:** Department of Medical Intensive Care Unit, The First Affiliated Hospital, Sun Yat-Sen University, No.58 Zhongshan Road 2, Guangzhou, 510080 Guangdong China

**Keywords:** Acute respiratory distress syndrome, FoxM1, Mesenchymal stem cells

## Abstract

**Background:**

Injury of alveolar epithelial cells and capillary endothelial cells is crucial in the pathogenesis of acute lung injury/acute respiratory distress syndrome (ALI/ARDS). Mesenchymal stem cells (MSCs) are a promising cell source for ALI/ARDS treatment. Overexpression of Fork head box protein M1 (FoxM1) facilitates MSC differentiation into alveolar type II (AT II) cells in vitro. Moreover, FoxM1 has been shown to repair the endothelial barrier. Therefore, this study explored whether overexpression of FoxM1 promotes the therapeutic effect of bone marrow-derived MSCs (BMSCs) on ARDS by differentiation of BMSCs into AT II cells or a paracrine mechanism.

**Methods:**

A septic ALI model was established in mice by intraperitoneal administration of lipopolysaccharide. The protective effect of BMSCs-FoxM1 on ALI was explored by detecting pathological variations in the lung, total protein concentration in bronchoalveolar lavage fluid (BALF), wet/dry (W/D) lung weight ratio, oxidative stress levels, cytokine levels, and retention of BMSCs in the lung. In addition, we assessed whether FoxM1 overexpression promoted the therapeutic effect of BMSCs on ALI/ARDS by differentiating into AT II cells using SPC^−/−^ mice. Furthermore, the protective effect of BMSCs-FoxM1 on lipopolysaccharide-induced endothelial cell (EC) injury was explored by detecting EC proliferation, apoptosis, scratch wounds, tube formation, permeability, and oxidative stress, and analyzing whether the Wnt/β-catenin pathway contributes to the regulatory mechanism in vitro using a pathway inhibitor.

**Results:**

Compared with BMSCs-Vector, treatment with BMSCs-FoxM1 significantly decreased the W/D lung weight ratio, total BALF protein level, lung injury score, oxidative stress, and cytokine levels. With the detected track of BMSCs-FoxM1, we observed a low residency rate and short duration of residency in the lung. Notably, SPC was not expressed in SPC^−/−^ mice injected with BMSCs-FoxM1. Furthermore, BMSCs-FoxM1 enhanced EC proliferation, migration, and tube formation; inhibited EC apoptosis and inflammation; and maintained vascular integrity through activation of the Wnt/β-catenin pathway, which was partially reversed by XAV-939.

**Conclusion:**

Overexpression of FoxM1 enhanced the therapeutic effect of BMSCs on ARDS, possibly through a paracrine mechanism rather than by promoting BMSC differentiation into AT II cells in vivo, and prevented LPS-induced EC barrier disruption partially through activating the Wnt/β-catenin signaling pathway in vitro.

**Supplementary Information:**

The online version contains supplementary material available at 10.1186/s13287-023-03240-8.

## Introduction

Injury of alveolar epithelial cells and capillary endothelial cells (ECs) is the main cause of the development of acute lung injury/acute respiratory distress syndrome (ALI/ARDS) [1]. Epithelial injury is particularly crucial for the development of ARDS because it contributes to fluid balance dysregulation and alveolar edema [2]. There are two main types of alveolar epithelium: alveolar type I (AT I) cells and alveolar type II (AT II) cells, among which AT I cells account for 40% but cover 95% of the alveolar surface [3]. Although AT II cells only cover 5% of the alveolar surface, they secrete pulmonary surfactant, regulate alveolar fluid clearance and lung innate immunity against infection, and contribute to epithelial repair after injury by proliferating and differentiating into AT I cells [4, 5]. Therefore, AT II cells play a more important role than AT I cells in protecting lungs from damage. Reducing impairment of the epithelium, especially AT II cells, would be an effective method to treat ARDS, including alleviation of apoptosis, oxidative stress, and inflammation of alveolar epithelial cells [6, 7]. In addition, lung endothelial barrier dysfunction is a primary underlying cause of sepsis-induced ALI [8]. Tight and adherens junctions of the EC barrier are disrupted, allowing protein, inflammatory cells, and fluid to enter the lungs, resulting in alveolar edema and lung dysfunction [9]. Thus, preserving the endothelial function and ensuring barrier integrity may be possible strategies for preventing and treating ARDS.

Mesenchymal stem cells (MSCs) are a type of stem cell with self-renewal, differentiation potential, and cytokine secretion capacity that have promising prospects for the treatment of various diseases [10, 11]. Recent studies have focused on the possibility of using MSCs for treatment of ARDS through their differentiation capacity. Most studies have genetically modified MSCs because of their low differentiation efficiency in vivo [12]. Han et al. [13] demonstrated that overexpression of E-Prostanoid 2 Receptor in MSCs increases their pulmonary residence and Attenuated Lung Injury. Similarly, Shao et al. [14] showed that CXCR7 facilitates the therapeutic effects of MSCs on acute lung injury (ALI) by increasing pulmonary homing and differentiation into AT II cells. We confirmed that overexpression of Forkhead box protein M1 (FoxM1) promotes differentiation of bone marrow-derived MSCs (BMSCs) into AT II cells via activation of the Wnt/β-catenin pathway in vitro [15]. However, the role of BMSCs in pulmonary epithelial cell repair remains controversial. It has been shown that it is a rare event for MSCs to migrate to the lungs and differentiate into epithelial cells [16]. Many preclinical studies have shown that MSCs modify important pathobiological pathways in ARDS and sepsis by releasing paracrine factors [17]. In addition, the therapeutic efficiency of MSCs can be enhanced by either genetic modification or engineering tools [18, 19]. Intriguingly, FoxM1 is also associated with endothelial injury in ALI. Huang et al. demonstrated that FoxM1 promotes rapid recovery and survival of endothelial barrier function in clinically relevant cecal ligation and puncture sepsis models [20]. Therefore, in this study, we explored the contribution of BMSCs overexpressing FoxM1 to the injury of alveolar epithelial and endothelial barriers in ARDS.

## Materials and methods

### BMSCs transduction and culture

BMSCs were derived from bone marrow of Sprague–Dawley (SD) rats acquired from the Animal Center of Sun Yat-sen University (Guangzhou, China). BMSCs were cultured with complete medium. Before transduction, BMSCs were characterized at passage three to assess their marker expression. The molecular markers of BMSCs were detected by flow cytometry [21]. The osteogenic, adipogenic, and chondrogenic differentiation capacities of BMSCs were detected by Alizarin red, Oil Red O, and Alcian blue staining. Isolation and culture of BMSCs were performed as previously described [22]. BMSCs in six-well culture plates (2 × 10^5^ cells/well) were transduced with viral supernatants at a multiplicity of infection (MOI) of 100 by adding 40 uL of transfection enhancement solution Hitrans G A (Shanghai, China). The medium was replaced after 24 h. After 72 h, successfully transfected cells were selected for subsequent experiments. Cells were used in experiments from passages three to ten.

### Preparation of experimental animals

Eight-week-old male C57BL/6 J mice were acquired from the Animal Center of Sun Yat-sen University (Guangzhou, China). Surfactant protein C (SPC) gene knockout (SPC^−/−^) mice were purchased from Cyagen Biosciences (Santa Clara, CA). Animal experiments were conducted as recommended by the Animal Care and Use Committee of the First Hospital of Sun Yat-sen University. Mouse LPS-ALI models were established by a previously described method [23, 24]. The LPS-ALI mouse model was established by intraperitoneal injection of lipopolysaccharide (LPS; 10 mg/kg, *Escherichia coli* 055:B5; Sigma-Aldrich, St. Louis, MO). C57BL/6 J and SPC^−/−^ mice (18–22 g body weight) were randomized into four groups (*n* = 12 per group): control group mice administered 100 uL of phosphate-buffered saline (PBS) via the tail vein and 100 uL PBS via intraperitoneal injection; LPS group mice received 100 uL PBS via their tail vein before LPS challenge; LPS + BMSCs-Vector group mice received BMSCs-Vector (1 × 10^6^ cells resuspended in 100 uL of PBS) by tail vein injection before LPS challenge; and LPS + BMSCs-FoxM1 group mice received BMSCs-FoxM1 (1 × 10^6^ cells in 100 uL PBS) by tail vein injection before LPS challenge. Body weights of mice were measured before and after LPS exposure. At 24 h post-LPS injection, animals were euthanized by intraperitoneal injection of pentobarbital sodium (180 mg/kg). Additionally, some SPC^−/−^mice were euthanized at 7 days post-LPS injection to obtain lung lobes for subsequent analyses. The Laboratory Animal Management Committee of Sun Yat-sen University Medicine Research Center approved this study.

### Lung wet-to-dry weight ratio

Right upper lungs were quickly resected. Blood was removed from the lung surface with distilled water and the lung surface was dried with filter paper. Weight was measured by a precision electronic balance (wet weight, W). To assess the degree of pulmonary oedema, lungs were incubated at 72 °C in an oven for 48 h to assess the dry weight (D) and calculate the lung W/D ratio.

### Protein levels in bronchoalveolar lavage fluid (BALF)

BALF was obtained by tracheal intubation with three flushes of 1 mL of pre-cooled PBS. After 10 min of centrifugation at 800× *g* and 4 °C, the total protein of BALF was measured by a bicinchoninic acid (BCA) assay kit (Beyotime, Haimen, China).

### Pulmonary histopathological examination

Left lungs were fixed in 4% paraformaldehyde, embedded in paraffin, cut into 3–4-μm-thick sections, and stained with hematoxylin and eosin (H&E; Beyotime). Microscopy (200× magnification) was used to assess histopathological variations in the lungs and evaluate lung injury scores. Lung scores were assessed by the following criteria: cellular infiltration, alveolar wall thickness, and hemorrhage, which were each scored from 0 to 4 (0, no injury; 1, injury in 25% of the field; 2, injury in 50%; 3, injury in 75%; 4, injury throughout the field) [25]. Areas were determined in a blinded manner in five equally spaced fields in each lung section. Counts of the average score for each lung section were summed and used as the ALI score.

### Masson staining

Masson trichrome staining was applied using a Trichrome Stain (Masson) Kit (Servicebio, Wuhan, China). Areas were determined in a blinded manner in five equally spaced fields in each lung section. The degree of fibrosis was evaluated as the integral optical density (IOD) using Image-Pro Plus [26].

### Evaluation of biochemical markers

According to the manufacturer’s instructions, a Malondialdehyde (MDA) Assay Kit (Beyotime), and Glutathione (GSH) and Superoxide Dismutase (SOD) Assay Kits (Nanjing Jiancheng, China) were used to detect MDA, GSH, and SOD, respectively.

### In vivo bioluminescence imaging

An IVIS® Kinetic system (Caliper, Hopkinton, MA, USA) was used for bioluminescence imaging to track BMSCs-FoxM1. After luciferin administration through the tail vein at the corresponding time point, anesthesia was induced using isoflurane. Imaging was performed on days 1, 2, 3, 4, 5, 7, and 8 for 10 min each time until sacrifice. Peak signals from a fixed region of interest were evaluated by Living Image® 4.0 software. Experiments were conducted in a blinded manner.

### Cryoimmunohistology

Tissue samples with fluorescent proteins were collected at designated time points, fixed, treated with a hypertonic sucrose solution, and placed flat in a frozen embedding box. Subsequently, an appropriate amount of OCT embedding agent was added to immerse the tissue. After soaking at 4 °C for 20–30 min, liquid nitrogen was used to snap freeze tissue. Tissue sections 10 µm in thickness were prepared on a Leica cryostat at − 20 °C, placed onto slides, dried, and then stored at − 20 °C for later analysis.

### Cell culture

EA.hy926 human umbilical vein ECs were obtained from Guangzhou Scissor Hand Gene Technology (Guangzhou, China) and maintained in complete medium and cultured in a standard incubator. The co-culture system was established in Transwell chambers with a 0.4-µm pore size (Corning, Corning, NY). BMSCs-Vector (1 × 10^5^ cells/mL) or BMSCs-FoxM1 (1 × 10^5^ cells/mL) was incubated in the upper chamber, and ECs (1 × 10^5^ cells/mL) were incubated in the lower chamber. Based on previous experiments and studies, LPS (150 ug/mL) was used simultaneously on ECs cocultured with BMSCs-Vector or BMSCs-FoxM1 for 24 h [18, 27]. Cells were divided into four groups: Control, LPS (150 ug/mL), LPS + BMSCs-Vector (150 ug/mL), and LPS + BMSCs-FoxM1 (150 ug/mL). To examine the protective effect of BMSCs-FoxM1 on LPS-induced EC injury via the Wnt/β-catenin pathway, a specific inhibitor of the pathway, XAV-939 (APExBIO, Houston, TX), was used. Based on previous experiments and studies, XAV-939 was applied to ECs at a concentration of 10 uM [18].

### In vitro scratch assay

ECs were seeded in six-well plates and grown to confluence. Monolayers were gently scratched using a 10-uL pipette tip to form a square scratch, washed thrice with 1 × PBS, and cultured with fresh serum-free medium for 24 h using the different co-culture systems described above. After 0 and 24 h, scratches were photographed microscopically with an automatic inverted fluorescence microscope (Leica, Wetzlar, Germany) and the percentage of wound healing was calculated as follows: wound healing percentage = [(original scratch area − final scratch area)/original scratch area] × 100%.

### 5-ethynyl-20-deoxyuridine (EdU) assay

Cell proliferation was detected using an EdU Kit (Ribobio, Guangzhou, China). Briefly, cells were incubated with EdU for 2 h and then fixed with 4% paraformaldehyde and permeabilized with 0.5% Triton X-100. Apollo® dye was then added and incubated in the dark for 30 min. Finally, cells were incubated with Hoechst 33,342 for 30 min and observed by fluorescence microscopy.

### Tube formation assay

Matrigel with reduced growth factors (Corning) was thawed at 4 °C and then used to coat 24-well plates (300 uL Matrigel at 37 °C per well) for 30 min. Next, the cell mixture (2 × 10^5^ cells/300 uL) was added to each Matrigel-coated well and the plate was incubated for 3 h at 37 °C in a 5% CO_2_ incubator. To improve visibility, Calcein AM fluorescent dye (5 uL; KeyGen, Nanjing, China) was added before fluorescence microscopy visualization. Finally, we randomly selected three fields of view for each well to quantify tube formation and count numbers of intersecting vessels.

### Apoptosis assays

Apoptosis was measured using annexin V-fluorescein isothiocyanate (AV-FITC)/propidium iodide (PI) (BD Biosciences, Franklin Lakes, NJ). EA.hy926 was plated in six-well plates at a density of 1 × 10^5^ cells/well and incubated until cells adhered to the wall. Subsequently, cells were cultured for 24 h using the various co-culture regimes described above. After being washed twice with cold PBS, cells were stained with Annexin V-FITC/PI in binding buffer in the dark at room temperature. Finally, cells were analyzed by using flow cytometry (Beckman Coulter, Brea, CA).

### Cell permeability assay

A Transwell permeability assay was used to assess the permeability of endothelial cells. Cells (2 × 10^5^) in 300 uL of medium were seeded in the membrane of each 6.5-mm Transwell insert to form a fusible monolayer. Next, cells were co-cultured in a 24-well plate using the various co-culture protocols described above. After 24 h, the EC-seeded chambers were transferred to new 24-well plates, the medium above the chambers was removed, and the chambers were refilled with medium containing horseradish streptavidin peroxidase. After 5 min of incubation, 20 uL of medium was transferred from the lower chamber to a new 96-well plate and add 50 uL of TMB substrate was added to each well. After 5–20 min of incubation at room temperature, 25 uL of stop solution (Sigma) was added to well. Finally, the absorbance at 450 nm of each well was assessed with a microplate reader.

### Enzyme linked immunosorbent assay (ELISA)

According to the manufacturer’s instructions, corresponding ELISA kits (eLGbio, China) were used to detect tumor necrosis factor α (TNF-α) and pro-inflammatory factors interleukin 1β (IL-1β), IL-6, IL-4, and IL-10.

### Western blot

Total protein extraction from lung tissues was conducted using radioimmunoprecipitation assay lysis buffer with PMSF. Total protein was quantified by BCA assay. Protein samples were separated on 10% sodium dodecyl sulfate–polyacrylamide gel electrophoresis gels, and transferred to polyvinylidene fluoride membranes. After 1 h of blocking with 5% dry skimmed milk at room temperature, membranes were probed with the following primary antibodies at a dilution of 1:1000: anti-prosurfactant protein B (SPB; Santa Cruz, Dalla, TX), anti-prosurfactant protein C (SPC; Abcam, Cambridge, UK), anti-FoxM1 (Proteintech, Rosemont, IL), anti-β-actin (DEWEIBIO, China), anti-β-catenin, anti-VE-cadherin, anti-BCL-2, and anti-BAX (Cell Signaling Technology, Danvers, MA) at 4 °C overnight. After washing three times with Tris-buffered saline containing Tween, membranes were incubated for 1 h using horseradish peroxidase-conjugated secondary antibodies at room temperature. The loading control was β-actin. Imaging was performed using a gel imager and evaluated using ImageJ software v1.4.0.

### Quantitative real-time polymerase chain reaction (qRT-PCR)

An RNA-Quick Purification kit (ESscience, China) was used to extract total RNA from lung tissue. RNA was quantified spectrophotometrically (Nanodrop ND-2000; Thermo Scientific, Waltham, MA) and reverse transcribed into cDNA using a NovoScript Plus All-In-One 1st Strand cDNA Synthesis SuperMix (NovoProtein, Shanghai, China). qRT-PCR employed a SYBR qPCR Mix (NovoProtein) and real-time fluorescence quantitative PCR system (Bio-Rad, Hercules, CA). Specific primers were synthesized by ThingKe Biotechnology (Guangzhou, China) and are listed in Table 1. Relative expression levels were calculated by normalizing expression of the GAPDH housekeeping gene using the 2^−ΔΔCt^ method and are presented as the fold increase compared with the control.

**Table 1:** Quantitative real-time polymerase chain reaction primers

Gene name	Forward primer (5′-3′)	Reverse primer (5′-3′)
Interleukin 1beta (IL-1β)	GTCGCTCAGGGTCACAAGAA	GTGCTGCCTAATGTCCCCTT
Interleukin 6 (IL-6)	TCTTCAACCAAGAGATAAGCTGGA	CGCACTAGGTTTGCCGAGTA
Interleukin 8 (IL-8)	TGTTCACAGGTGACTGCTCC	AGCCCATAGTGGAGTGGGAT
Transforming growth factor beta (TGF-β)	CTGCTGACCCCCACTGATAC	GGGCTGATCCCGTTGATTTC
Macrophage Inflammatory Protein 1 Alpha (MIP-1α)	TGCCAAGTAGCCACATCGAG	GAGATGGGGGTTGAGGAACG
GAPDH	CAGTGGCAAAGTGGAGATTGTTG	TCGCTCCTGGAAGATGGTGAT

### Statistical analysis

Data are presented as the mean ± standard error of the mean (SEM). Results were evaluated using GraphPad Prism 8 (GraphPad, San Diego, CA). Independent Student’s t-tests were used for comparisons of two groups, and one-way ANOVA followed by Tukey’s test was used for comparisons of multiple groups*. p* < 0.05 denoted significance.

## Results

### Characterization of BMSCs

**Fig. 1:**
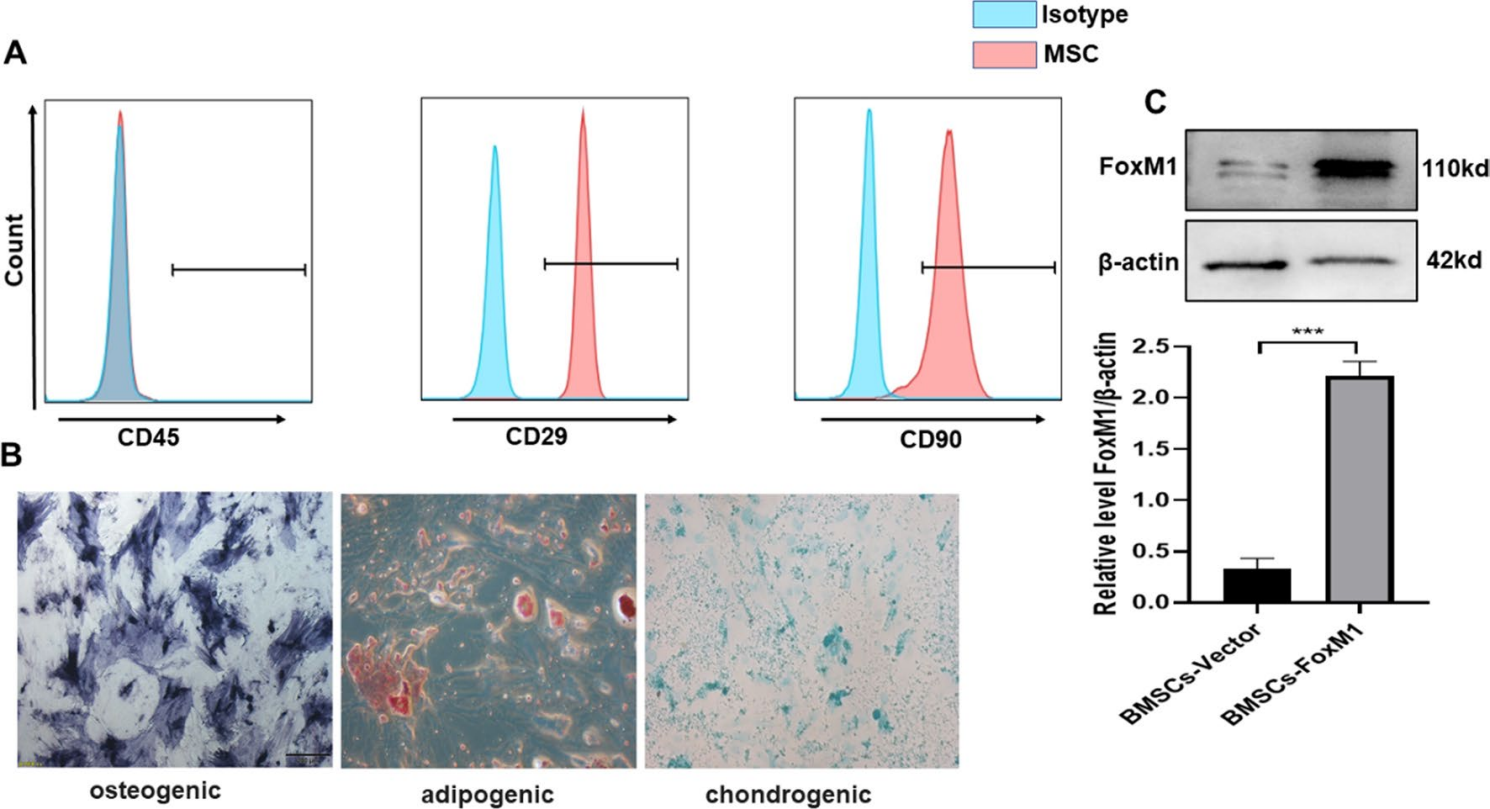
Isolation and characterization of rat marrow-derived MSCs. **A** Histograms of flow cytometry analysis showing positive expression of MSCs markers CD45, CD29, CD90, and control having unlabeled cells. **B** Multilineage differentiation capacity (osteogenic, adipogenic, and chondrogenic differentiation) of MSCs (100×). **C** Determination of FoxM1 protein expressions by western blot analysis. Values are expressed as mean ± SEM, *n* = 3, *P < 0.05. The blots were cropped and these uncropped images placed in Additional file 1: Fig. S1

The morphological features and molecular markers of BMSCs were identified. Flow cytometry results showed that the surface of BMSCs positively expressed CD90 and CD29, but were negative for CD45 (Fig. 1A). In addition, osteogenic, adipogenic, and chondrogenic differentiation of BMSCs could be detected by Alizarin Red, Oil Red O, and Alcian blue staining (Fig. 1B). As indicated in Fig. 1C, FoxM1 expression was significantly increased in BMSCs-FoxM1 compared with BMSCs-Vector, indicating that BMSCs infected by FoxM1 lentivirus had significantly increased FoxM1 expression.

### BMSCs overexpressing FoxM1 ameliorate LPS-mediated lung injury

**Fig. 2:**
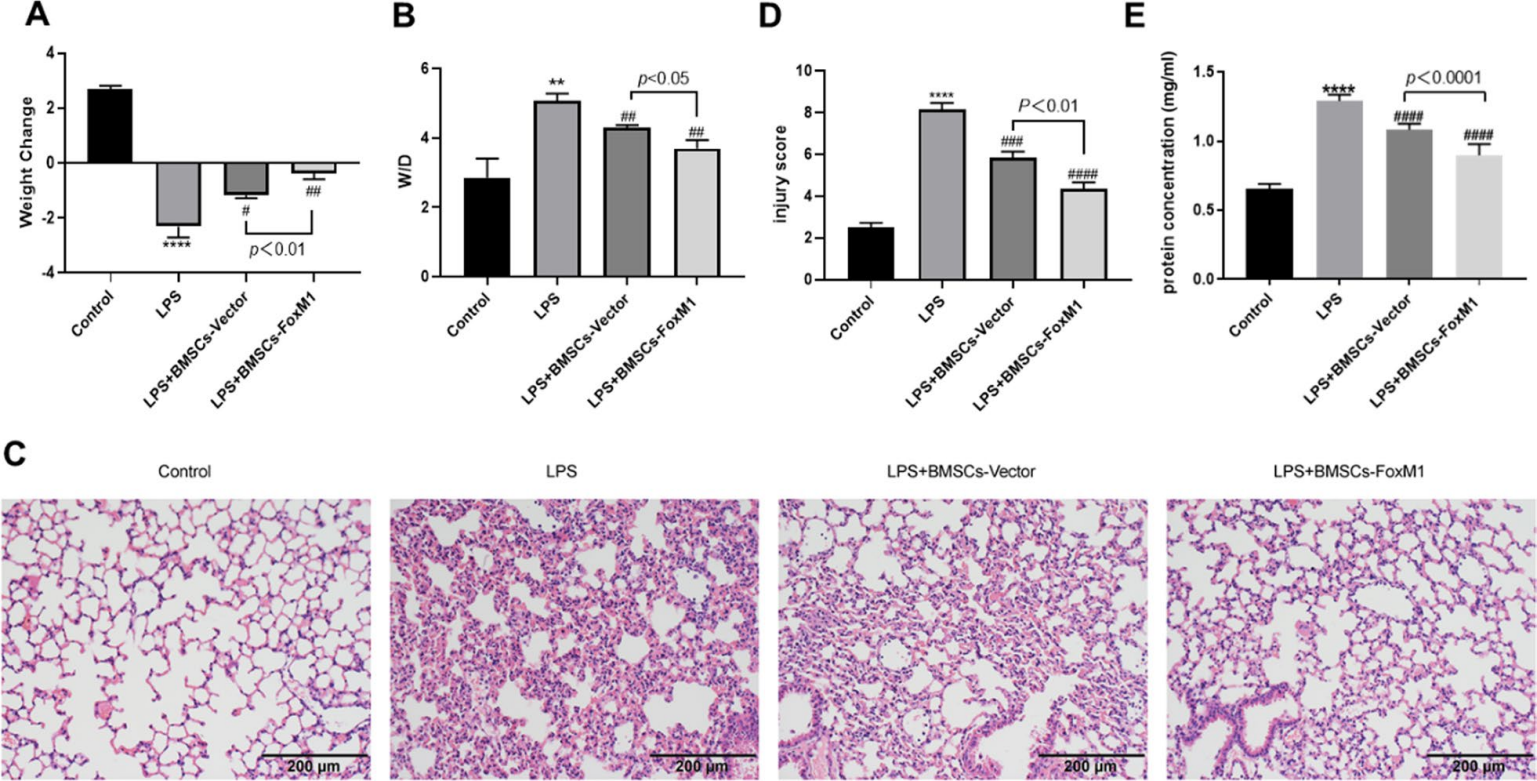
Effect of BMSCs overexpressing FoxM1 on LPS-induced ALI in mice. **A** Effects of BMSCs overexpressing FoxM1 on body weight. **B** Lung wet/dry ratio of mice with various treatment was shown. **C** & **D** Lung tissues were detected by HE staining to evaluate the severity of lung injury, magnification 200×. **E** Total protein concentration in BALF was assessed with a BCA Protein Concentration Assay Kit. Values are expressed as mean ± SEM. (*n* = 6, *Compared with control group, *p < 0.05, **p < 0.01, ***p < 0.001; #compared with LPS group, ^#^*p* < 0.05, ^##^*p* < 0.01, ^###^*p* < 0.001)

Figure 2A shows that, relative to the control group, body weights of LPS, LPS + BMSCs-Vector, and LPS + BMSCs-FoxM1 groups were significantly decreased. Relative to the LPS group, body weights of LPS + BMSCs-Vector and LPS + BMSCs-FoxM1 groups were significantly increased. These effects were larger in the LPS + BMSCs-FoxM1 group relative to the LPS + BMSCs-Vector group. As shown in Fig. 2B, the W/D weight ratio of lung tissue samples from the LPS group was markedly higher relative to the control group. The W/D weight ratio of lung tissue samples from LPS + BMSCs-FoxM1 and LPS + BMSCs-Vector groups was markedly lower than that of lung tissue samples from the LPS group, and significantly lower in the LPS + BMSCs-FoxM1 group compared with the LPS + BMSCs-Vector group. HE staining used to assess pathological alterations in lung tissues at 24 h after LPS treatment showed that the LPS group had notable morphological variations, such as inflammatory cell infiltration and oedema with hemorrhaging, relative to the control group (Fig. 2C). However, pretreatment with BMSCs-Vector or BMSCs-FoxM1 alleviated these pathological alterations in lungs, and the LPS + BMSCs-FoxM1 group had improved pathological changes of lung tissue relative to the LPS + BMSCs-Vector group. Lung injury scores were markedly higher in the LPS group, whereas BMSCs-FoxM1 treatment significantly reduced these scores (Fig. 2D). Additionally, total BALF protein levels were elevated in the LPS group, decreased in BMSCs-Vector and BMSCs-FoxM1 groups, and even more significantly decreased in the LPS + BMSCs-FoxM1 group, suggesting a reduction of alveolar protein leakage (Fig. 2E). These findings suggest that BMSCs overexpressing FoxM1 relieved LPS-induced lung injury.

### Protective effects of BMSCs overexpressing FoxM1 against oxidative stress in ALI mice

**Fig. 3:**
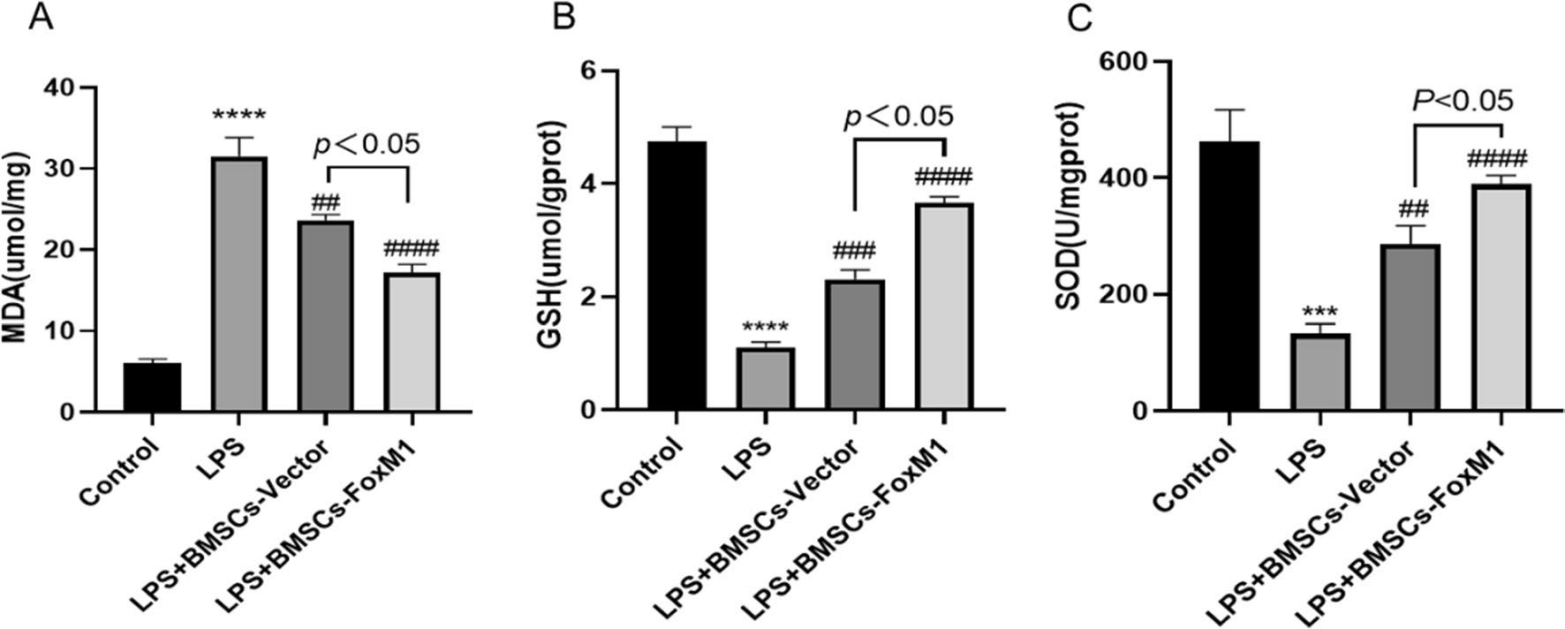
BMSCs overexpressing FoxM1 inhibited LPS-induced oxidative stress in lung tissues. **A** MDA in lung tissue. **B** GSH activity. **C** SOD activity. Values are expressed as mean ± SEM. (*n* = 6, *Compared with control group, *p < 0.05, **p < 0.01, ***p < 0.001; #compared with LPS group, ^#^*p* < 0.05, ^##^*p* < 0.01, ^###^*p* < 0.001)

Oxidative stress can lead to lung destruction. Therefore, the oxidative status of lung tissue was evaluated by assessing MDA, GSH, and SOD levels as indicated in Fig. 3. Relative to the control group, MDA levels were much higher, while GSH and SOD activities were dramatically lower after intraperitoneal injection of LPS. Pretreatment with BMSCs-Vector or BMSCs-FoxM1 profoundly decreased the MDA content and increased GSH and SOD levels relative to the LPS group. Additionally, in terms of alleviating MDA content and activities of GSH and SOD, BMSCs-FoxM1 was more effective than BMSCs-Vector. These results suggest that BMSCs-FoxM1 pretreatment markedly inhibited oxidative stress by enhancing antioxidant enzyme activities of lung tissue in LPS-induced ALI.

### Effects of BMSCs that overexpress FoxM1 on inflammatory cytokine content in lung tissue

**Fig. 4:**
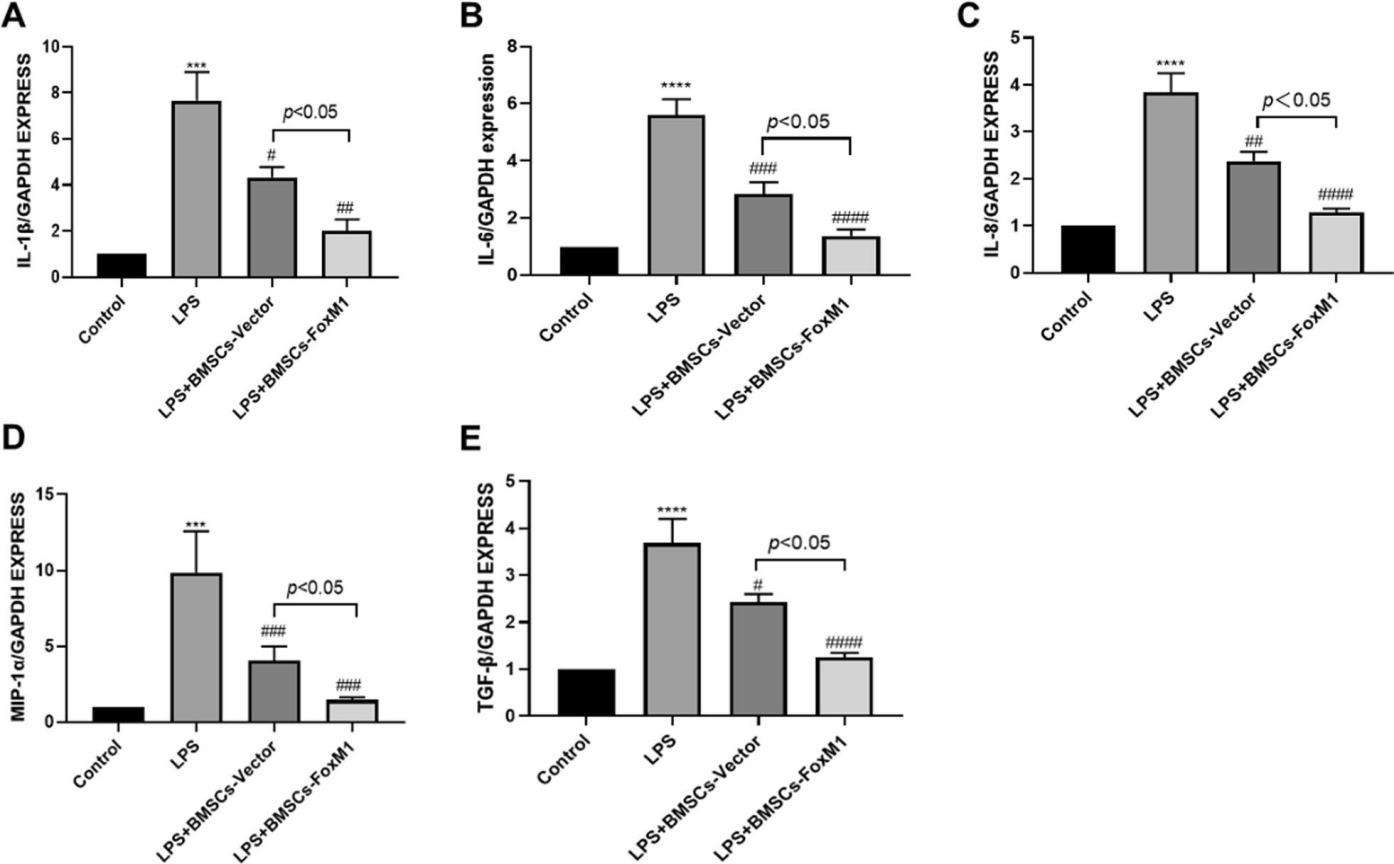
BMSCs overexpressing FoxM1 decreased inflammatory cytokine expression in the LPS-induced acute lung injury. **A **IL-1β, **B **IL-6, **C** IL-8, **D** MIP-1α, **E** and TGF-β. Values are expressed as mean ± SEM. (*n* = 6, *Compared with control group, *p < 0.05, **p < 0.01, ***p < 0.001; #compared with LPS group, ^#^*p* < 0.05, ^##^*p* < 0.01, ^###^*p* < 0.001)

Various proinflammatory factors are involved in inflammatory reactions in ALI. Thus, we assessed IL-1β, IL-6, IL-8, MIP-1α, and TGF-β levels in lung tissue as indicated in Fig. 4. Relative to the control group, the LPS group displayed marked elevations of IL-1β, IL-6, MIP-1α IL-8, and TGF-β. Moreover, levels of IL-1β, MIP-1α, IL-6, IL-8, and TGF-β were markedly decreased in LPS + BMSCs-Vector and LPS + BMSCs-FoxM1 groups relative to the LPS group, and markedly decreased in the LPS + BMSCs-FoxM1 group relative to the LPS + BMSCs-Vector group.

### BMSCs overexpressing FoxM1 reduced lung fibrosis

**Fig. 5:**
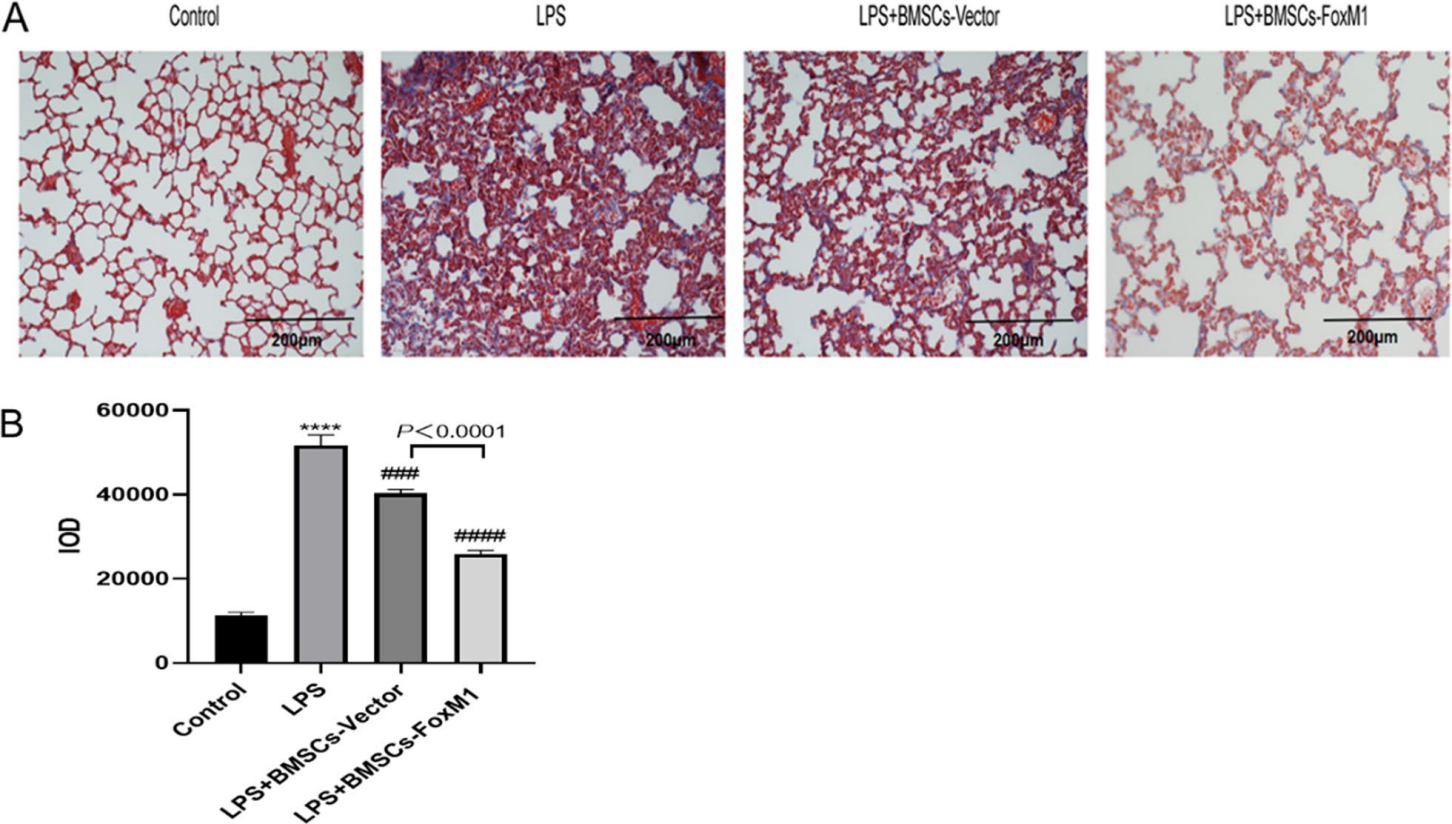
**A** Pulmonary fibrosis, assessed by Masson trichrome, stained in blue was greater in the lungs after LPS group, magnification 200× . **B** Quantitative analysis of pulmonary fibrosis. Values are expressed as mean ± SEM. (*n* = 6, *Compared with control group, *p < 0.05, **p < 0.01, ***p < 0.001; #compared with LPS group, ^#^*p* < 0.05, ^##^*p* < 0.01, ^###^*p* < 0.001)

To evaluate pulmonary fibrosis, collagen depositions in lung tissue 24 h after LPS stimulation were assessed by Masson’s trichrome staining. The results revealed significantly elevated collagen levels in the LPS group relative to the control group. Pulmonary fibrosis was significantly lower in LPS + BMSCs-Vector and LPS + BMSCs-FoxM1 groups relative to the LPS group. Moreover, decreased lung fibrosis was noted in the LPS + BMSCs-FoxM1 group relative to the LPS + BMSCs-Vector group (Fig. 5).

### Residence of BMSCs-FoxM1 in the lungs

**Fig. 6:**
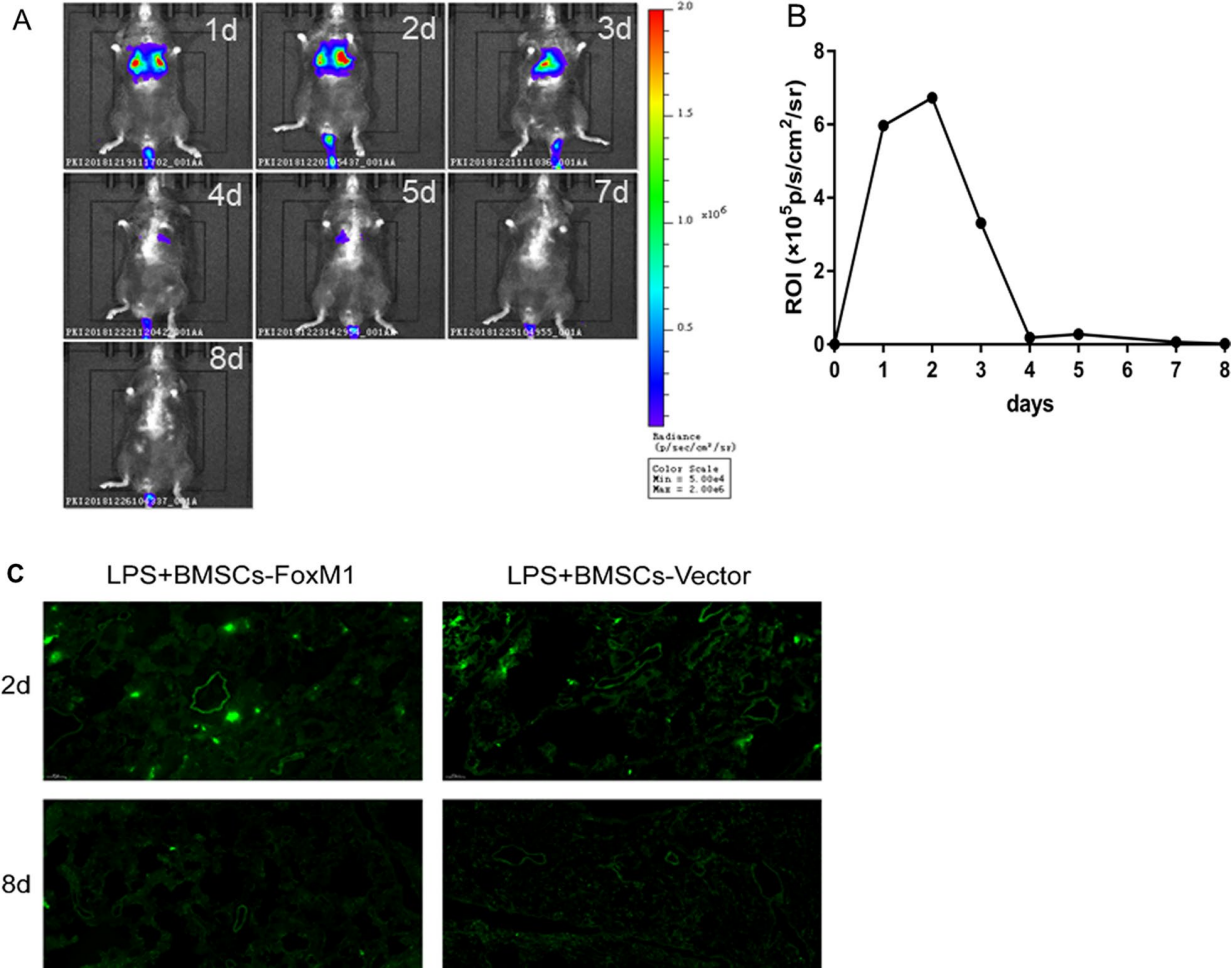
The graft retention of BMSCs in the lungs after LPS challenge. **A** In vivo bioluminescence imaging of injured lungs from mice at 1, 2, 3, 4, 5, 7and 8 days after BMSCs transplantation. **B** The fluorescence intensity of lung ROI at different time; ROI, Region of Interest. **C** Observation of frozen sections of lung tissue after injection of BMSCs carrying green fluorescent protein through the tail vein of mice (10×)

To investigate whether overexpression FoxM1 promoted the differentiation of BMSCs into AT II cells, in vivo bioluminescence imaging was performed on lungs 1, 2, 3, 4, 5, 7, and 8 days after BMSCs-FoxM1 implantation to track intrapulmonary BMSCs-FoxM1. The results showed that retention of BMSCs-FoxM1 in the lungs peaked within 1–2 days after BMSCs-FoxM1 implantation, but essentially disappeared within 4–7 days (Fig. 6A, B). We further observed green fluorescent protein (GFP) in BMSCs by preparing frozen sections of lung tissue. More GFP was observed in lungs of the ALI group on the second day after BMSCs-FoxM1 or BMSCs-Vector implantation, but GFP had almost disappeared by 8 days (Fig. 6C).

### Overexpression of FoxM1 does not induce BMSCs to differentiate into AT II cells

**Fig. 7:**
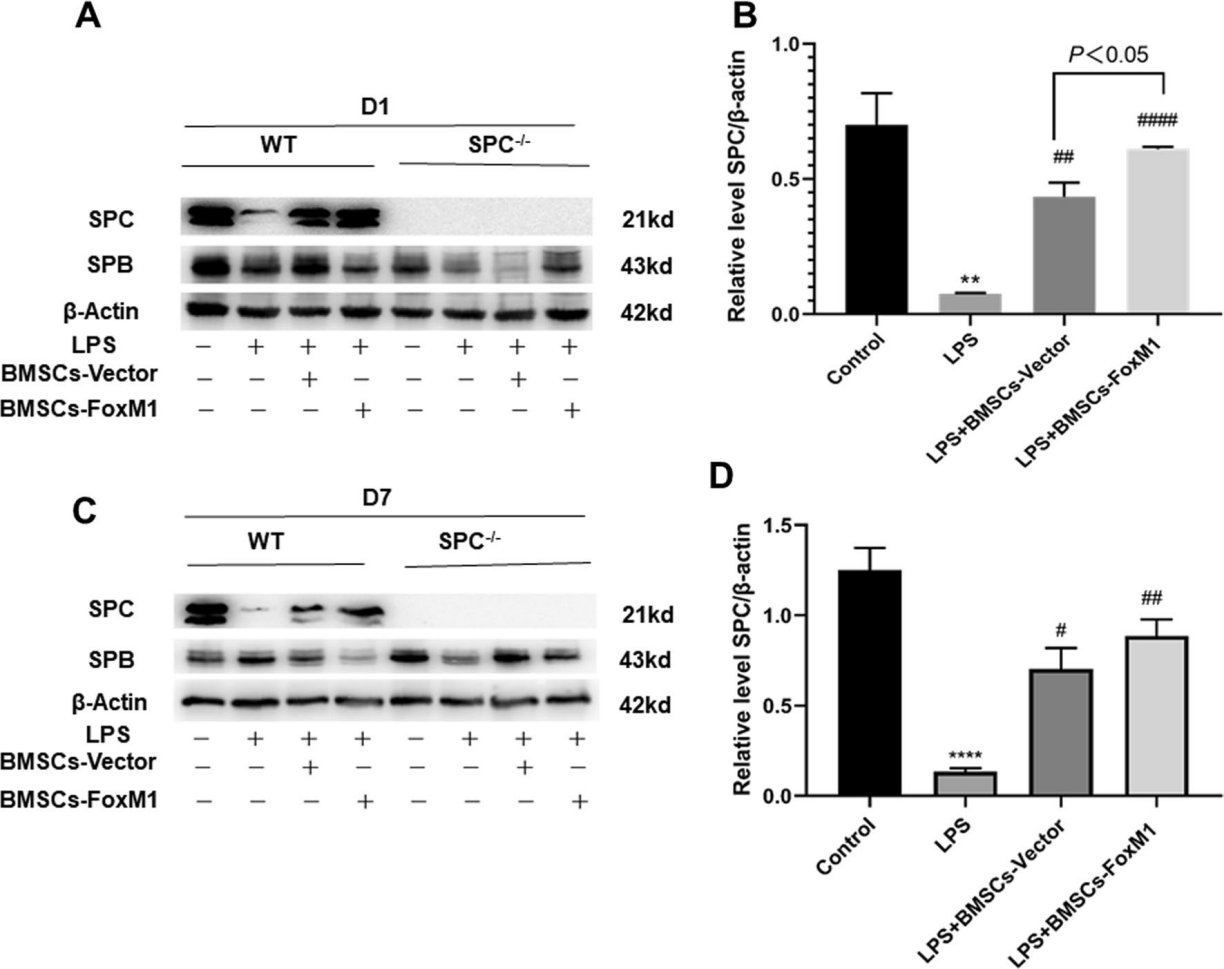
Effects of Overexpression of FoxM1 on protein expressions of SPB, and SPC in lung tissue of LPS-induced ALI. **A** Western blot showing changes in the SPB and SPC protein expression in lung tissues from mice 24 h post-LPS. **B** Quantitative analysis of SPC expression in lung tissues from mice 24 h post-LPS. **C** Western blot showed the changes of SPB and SPC protein expression in lung tissues from mice 7 days post-LPS. **D** Quantitative analysis of SPC expression in lung tissues from mice 7 days post-LPS. Values are expressed as mean ± SEM. (*n* = 3, *Compared with control group, *p < 0.05, **p < 0.01, ***p < 0.001; #compared with LPS group, ^#^*p* < 0.05, ^##^*p* < 0.01, ^###^*p* < 0.001). The blots were cropped and these uncropped images placed in Additional file 1: Fig. S7

To investigate whether FoxM1 overexpression promoted the differentiation of BMSCs into AT II cells in vivo, we verified the expression of SPC protein in SPC^−/−^ and WT mice by western blotting. At 1 day after BMSCs implantation, SPC content was markedly increased in LPS + BMSCs-Vector and LPS + BMSCs-FoxM1 groups relative to the LPS group. BMSCs overexpressing FoxM1 further increased SPC levels in lungs relative to the LPS + BMSCs-Vector group (Fig. 7A, B). Moreover, comparable findings were obtained 7 days after BMSCs implantation (Fig. 7C, D). However, at 1 and 7 days after BMSCs implantation, SPC was not expressed in LPS + BMSCs-Vector or LPS + BMSCs-FoxM1 groups of SPC^−/−^ mice, indicating that the BMSCs might not have differentiated into AT II cells (Fig. 7A, B). There were no marked differences in SPB expression among groups, regardless of genotype (WT or SPC^−/−^) (Fig. 7C, D). These findings suggest that FoxM1 overexpression enhanced the therapeutic effect of BMSCs on ALI/ARDS**,** possibly through a paracrine mechanism rather than by promoting BMSC differentiation into AT II cells in vivo.

### BMSCs overexpressing FoxM1 promote EC proliferation, migration, and tube formation ability in vitro

**Fig. 8:**
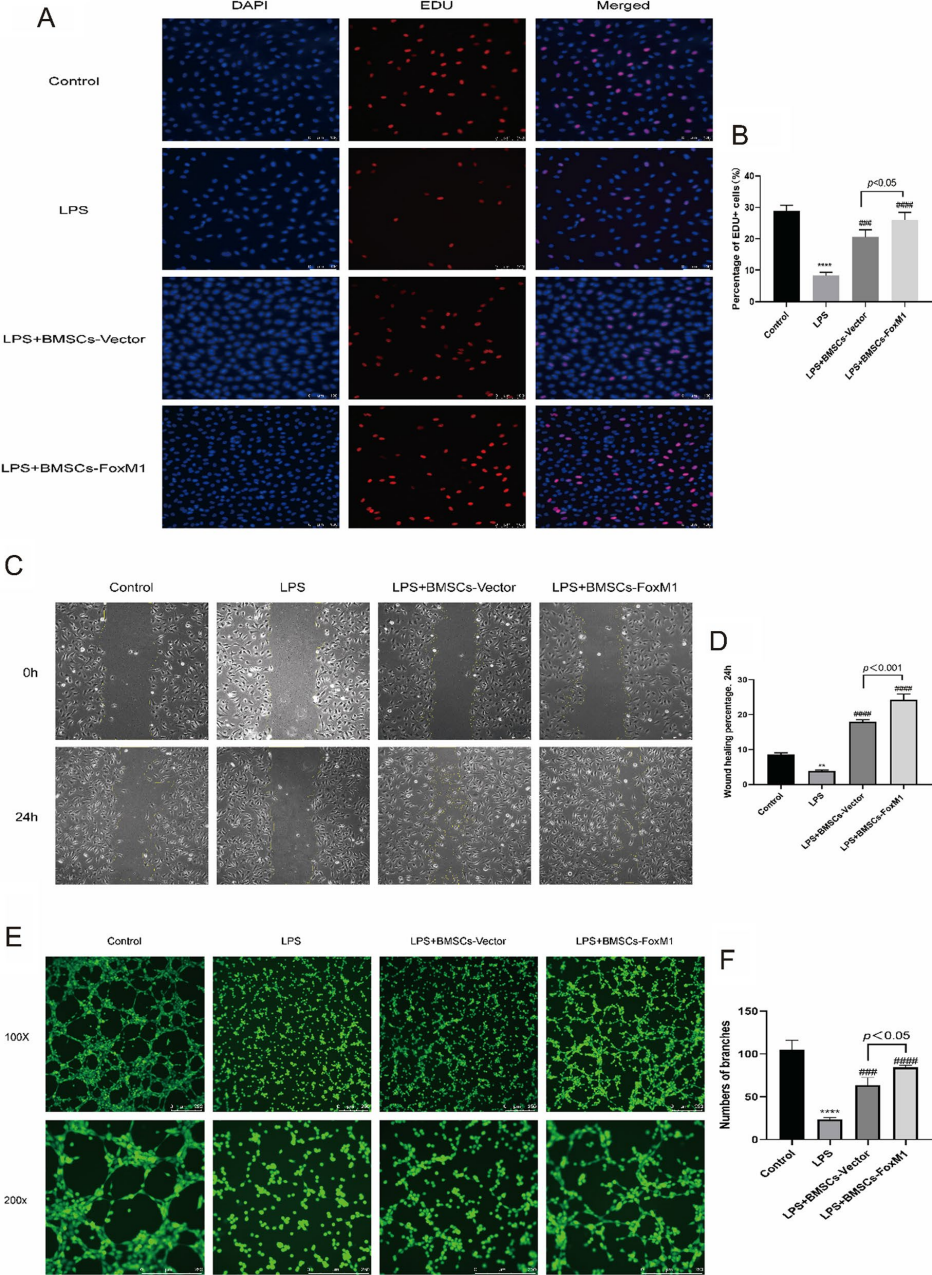
BMSCs-FoxM1 coculture promotes the proliferation, migration, and tube formation ability of ECs. **A** EdU was measured for EC proliferative capacity, of which the blue color indicated the nuclear localization and the red color indicated the proliferation-active cells (magnification 100×) **B** Quantitative analysis was conducted by calculating the percentage of proliferation-active cells, and the results suggested that BMSCs-FoxM1 coculture dramatically promoted EC proliferation capacity after LPS-induced injury compared to BMSCs-Vector coculture groups. **C** The scratch assay was conducted to assess the migration capability of EC, and representative images of the scratches at different time points at 0 h and 24 h are shown (magnification 100×). **D** Quantitative analysis of the changes in the scratched areas was performed using Image J software, and results suggested that the migration ability of ECs was significantly increased in the BMSCs-FoxM1 coculture group compared to BMSCs-Vector coculture group. **E** Tube formation assay was performed to detect EC angiogenic capacity, and Calcein AM fluorescent dye was used to enhance the visibility of tube and network formation in Matrigel (magnification 100× , 200×), along with the trajectories of tubes and networks were also depicted accordingly. **F** Quantitative analysis suggested that coculture with BMSCs-FoxM1 significantly promoted the EC tube formation ability compared to that in BMSCs-Vector coculture group. Values are expressed as mean ± SEM. (*n* = 3, *Compared with control group, *p < 0.05, **p < 0.01, ***p < 0.001; #compared with LPS group, #*p* < 0.05, ##*p* < 0.01, ###*p* < 0.001)

ECs were exposed to LPS in vitro to mimic vascular endothelial cell injury during sepsis-induced lung injury. Because EC proliferation plays a significant role in endothelial barrier repair [28–32], we used an EdU assay to assess the proliferative capacity of ECs. The results showed that the proliferation ability of ECs exposed to LPS was significantly decreased compared with the control group, but increased after co-culture with BMSCs. However, co-culture with BMSCs overexpressing FoxM1 yielded an increased protective effect (Fig. 8A, B). Moreover, the migratory ability of ECs is also related to endothelial regeneration [31, 33]. Hence, to determine whether BMSCs overexpressing FoxM1 enhanced the mobility of ECs, we quantified their migration. The results show that co-culture with different BMSCs increased migration relative to the LPS group. Compared with the BMSCs-Vector group, the migration capacity of ECs was markedly increased in the BMSCs-FoxM1 group (Fig. 8C, D). Finally, to evaluate another aspect of endothelial repair, we assessed the effect of BMSCs-FoxM1 on the lumen-forming ability of ECs in a test-tube formation assay. As indicated in Fig. 8E and F, the LPS group had almost no tube formation compared with the control group. Moreover, co-culture with different groups of BMSCs promoted EC tube formation ability, markedly in the LPS + BMSCs-FoxM1 group.

### BMSCs overexpressing FoxM1 attenuated oxidative stress, apoptosis, and permeability of ECs

**Fig. 9:**
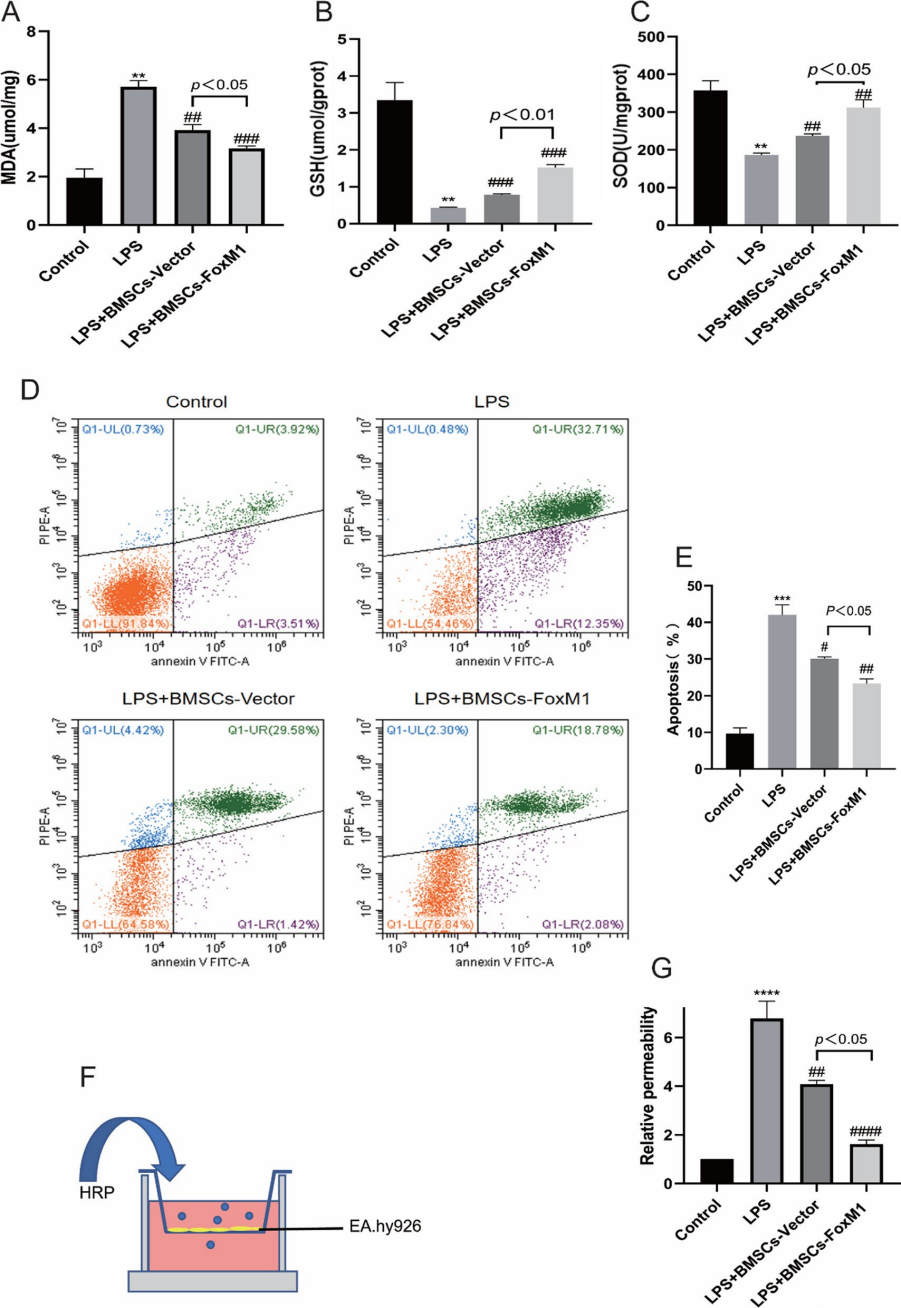
BMSCs-FoxM1 coculture attenuates oxidative stress, apoptosis and vascular permeability of ECs. **A** Levels of MDA in ECs. **B** Levels of GSH in ECs. **C** Levels of SOD in ECs. **D** Annexin V-FITC/PI was used to detect EC apoptosis. **E** Apoptosis ratio (%) via Annexin V and PI staining plus flow cytometry. **F** Transwell permeability assay was used to investigate the permeability of ECs. **G** Quantitative analysis of the changes of permeability of ECs. Values are expressed as mean ± SEM. (*n* = 3, *Compared with control group, *p < 0.05, **p < 0.01, ***p < 0.001; #compared with LPS group, #*p* < 0.05, ##*p* < 0.01, ###*p* < 0.001)

Oxidative stress plays a pivotal role in endothelial dysfunction. Therefore, the oxidative status of lung tissue was evaluated by assessing MDA, GSH, and SOD levels as indicated in Fig. 9A–C. Relative to the control group, MDA levels were much higher and activities of GSH and SOD were dramatically lower after LPS stimulation. Co-culture with BMSCs-Vector or BMSCs-FoxM1 profoundly decreased MDA contents and increased levels of GSH and SOD relative to the LPS group. Additionally, in terms of alleviating MDA content and activities of GSH and SOD, BMSCs-FoxM1 was more effective than BMSCs-Vector. These results suggest that BMSCs-FoxM1 pretreatment markedly inhibited oxidative stress by enhancing the antioxidant enzyme activities of LPS-induced ALI.

To investigate the effect of BMSCs-FoxM1 on LPS-induced apoptosis, we used AV-FITC/PI assays to detect apoptosis. We found that that rate of apoptosis increased following LPS exposure, relative to the control group. Co-culture with different group of BMSCs decreased apoptosis of ECs, and cell apoptosis was significantly inhibited by BMSCs-FoxM1 compared with the BMSCs-Vector (Fig. 9D, E). Furthermore, to investigate the effects of BMSCs-FoxM1 on EC barrier regulation, endothelial permeability was evaluated using an endothelial cell leakage assay (Fig. 9F). The results showed that LPS tended to increase permeability of the EC monolayer. Co-culture with different BMSCs greatly decreased EC permeability after LPS stimulation, and the BMSCs-FoxM1 group was markedly decreased relative to the BMSCs-Vector group (Fig. 9G).

### Involvement of Wnt/β-catenin signaling in protection against LPS-induced EC injury by co-culture with BMSCs overexpressing FoxM1

**Fig. 10:**
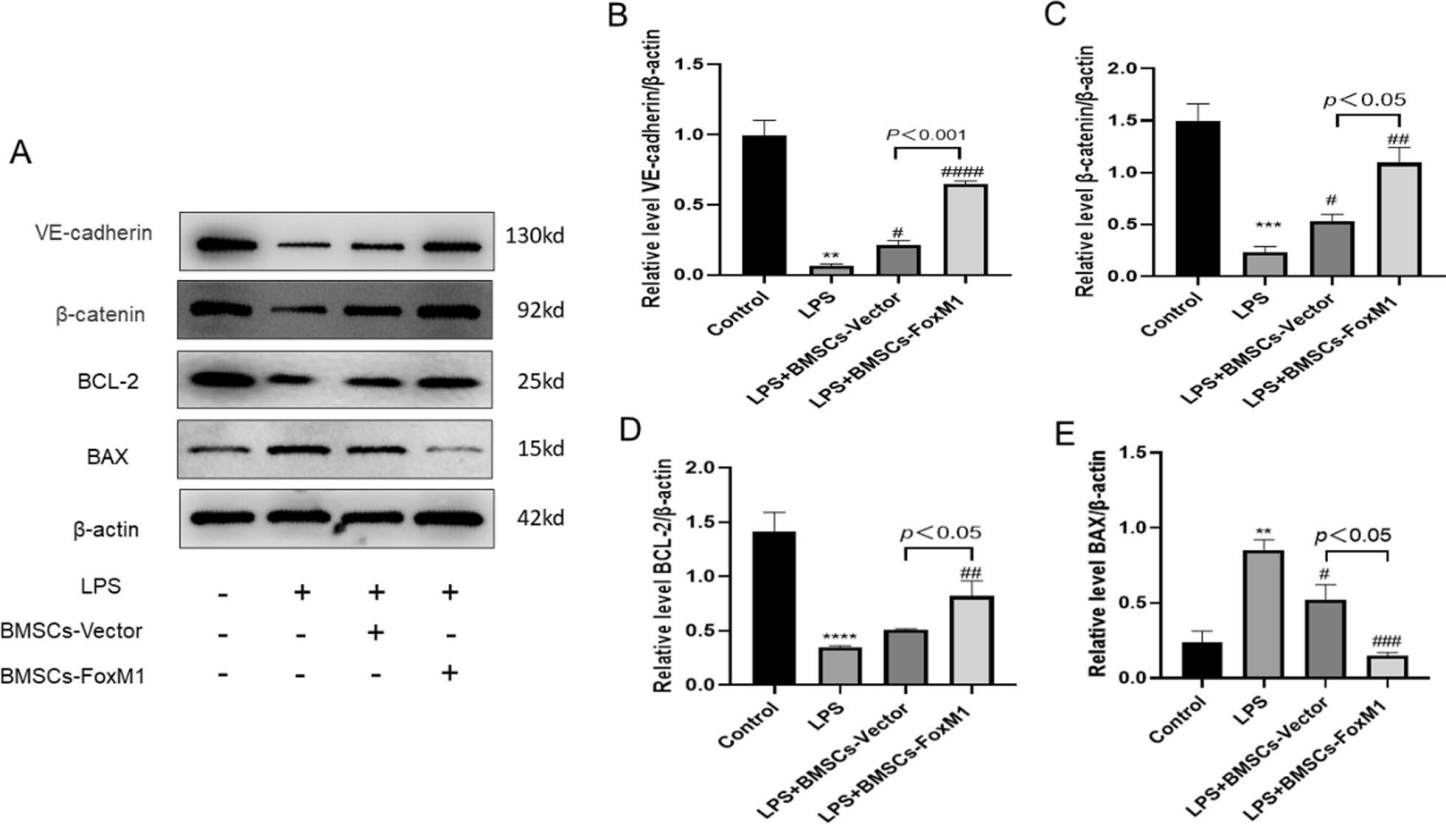
BMSCs-FoxM1 coculture protects against LPS-induced EC injury by activating the Wnt/β-catenin pathway. **A** Western blotting evaluated the expression of Wnt/β-catenin, VE-cadherin and apoptosis-related proteins (BCL-2, BAX). **B**–**E** Densitometric analysis of Western blots. β-actin served as an internal reference. Values are expressed as mean ± SEM. (*n* = 3, *Compared with control group, *p < 0.05, **p < 0.01, ***p < 0.001; #compared with LPS group, #*p* < 0.05, ##*p* < 0.01, ###*p* < 0.001). The blots were cropped and these uncropped images placed in Additional file 1: Fig. S10

**Fig. 11:**
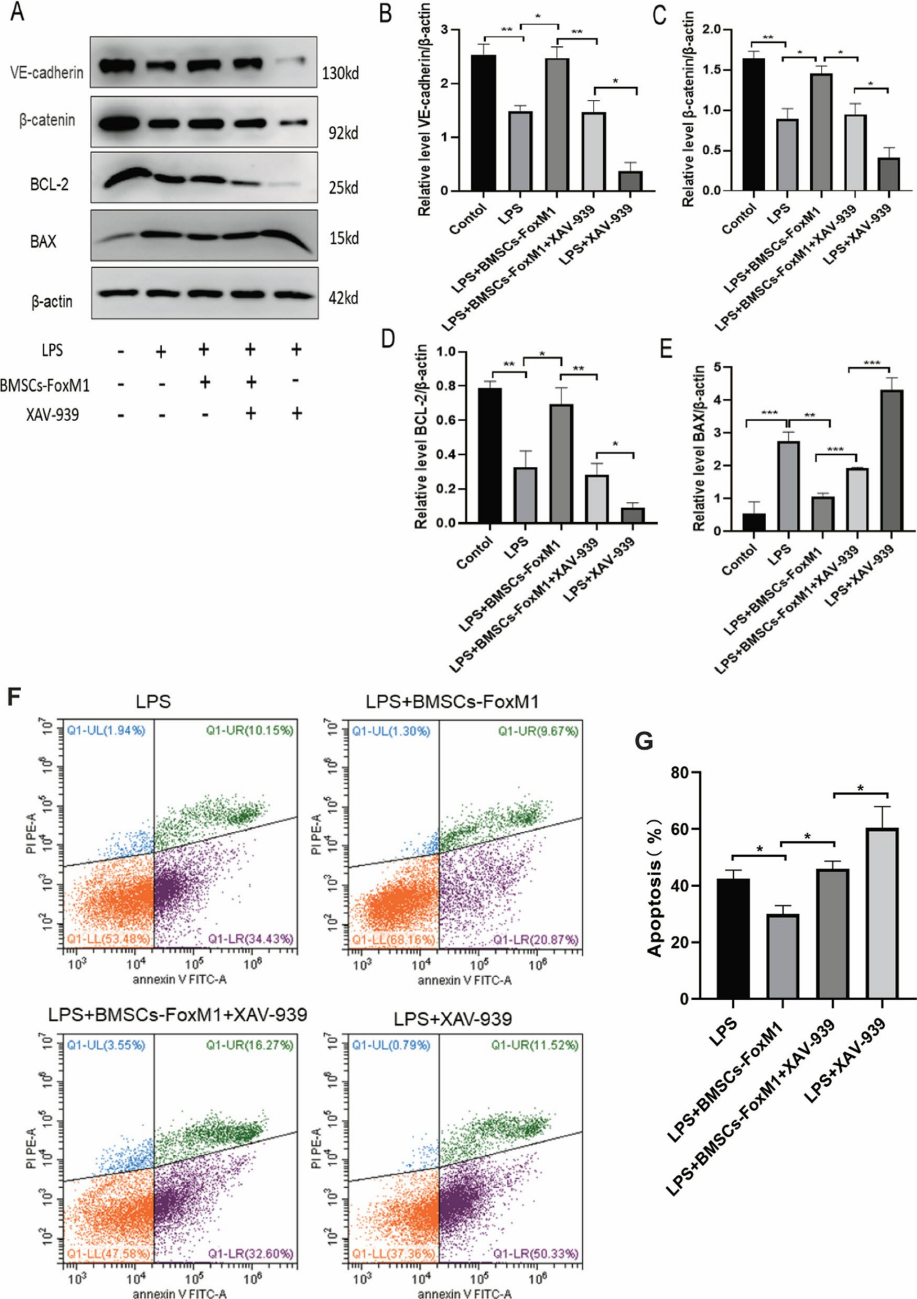
The effect of XAV-939 on BMSCs-FoxM1 coculture-induced changes in the Wnt/β-catenin pathway and apoptosis. **A** Exploring the effect of XAV-939 on BMSCs-FoxM1 coculture-induced changes in the Wnt/β-catenin pathway using Western blotting. **B**–**E** Densitometric analysis of Western blots. β-actin served as an internal reference. **F** Annexin V-FITC/PI was used to detect EC apoptosis. **G** Apoptosis ratio (%) via Annexin V and PI staining plus flow cytometry. Values are expressed as mean ± SEM. (*n* = 3, *p < 0.05, **p < 0.01, ***p < 0.001). The blots were cropped and these uncropped images placed in Additional file 1: Fig. S11

The protective mechanism of BMSCs-FoxM1 on ECs was further assessed by detecting levels of the β-catenin, VE-cadherin, BCL-2, and BAX proteins (Fig. 10A). We found that protein levels of VE-cadherin, β-catenin and anti-apoptotic protein BCL-2 were significantly decreased, while apoptosis-associated proteins BAX increased in ECs of the LPS group compared with the control group. Moreover, expression of β-catenin and VE-cadherin was significantly increased, the protein level of anti-apoptotic protein BCL-2 was considerably increased, and levels of apoptosis-associated proteins BAX were significantly decreased in the BMSCs-FoxM1 group compared with the LPS and BMSCs-Vector group (Fig. 10B–E). Intriguingly, XAV-939 reversed these changes in protein levels by decreasing Wnt/β-Catenin, VE-cadherin, and BCL-2 levels, and increasing levels of BAX (Fig. 11A–E). Meanwhile, flow cytometry results to detect apoptosis showed that the rate of apoptosis was reversed by XAV939 (Fig. 11F, G). These results indicate that BMSCs-FoxM1 may affect LPS-induced ALI by regulating the Wnt/β-catenin signaling pathway.

### BMSCs overexpressing FoxM1 modulate the cytokine milieu

**Fig. 12:**
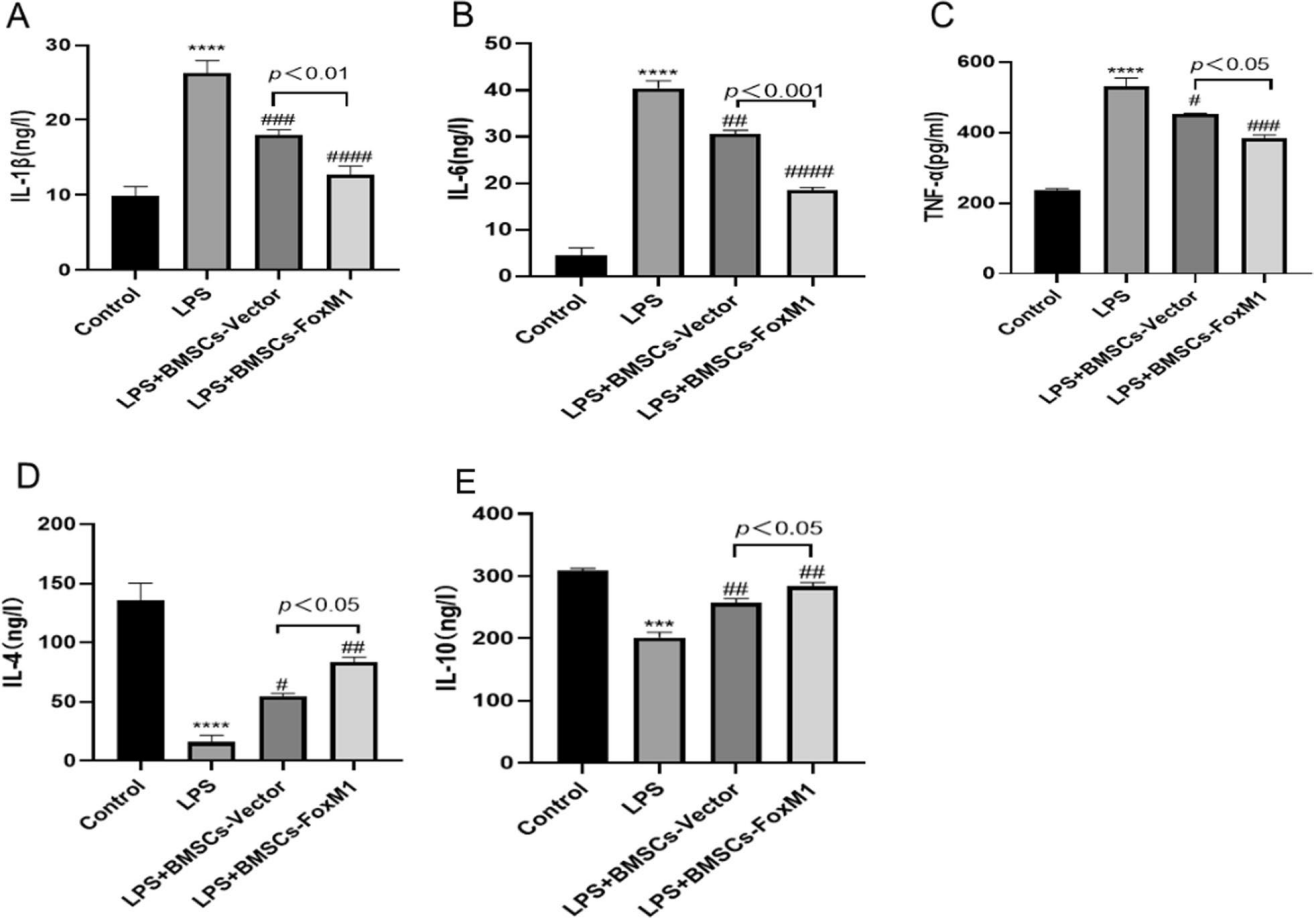
BMSCs-FoxM1 modulated LPS-induced inflammation in the ECs culture medium by ELISA. **A**, **B**, **C** BMSCs-FoxM1 decreased LPS-induced production of the EC-derived pro-inflammatory factors IL-1β(A), IL-6(B), and TNF-α(C) in the supernatants of the co-culture system. **D**, **E** BMSCs-FoxM1 suppressed the decrease of LPS-induced production of the EC-derived pro-anti-inflammatory IL-4(D), and IL-10(E) in the supernatants of the co-culture system. Values are expressed as mean ± SEM. (*n* = 3, *Compared with control group, *p < 0.05, **p < 0.01, ***p < 0.001; #compared with LPS group, #*p* < 0.05, ##*p* < 0.01, ###*p* < 0.001)

Inflammatory markers in cell culture media were detected by ELISA (Fig. 12). Compared with the LPS group, levels of IL-1β, IL-6, and TNF-α were decreased in BMSCs-FoxM1 and BMSCs-Vector groups. The observed decreases in IL-1β, IL-6, and TNF-α were larger in the BMSCs-FoxM1 group than the BMSCs-Vector group, while IL-4 and IL-10 increased relative to decreases in IL-1β, IL-6, and TNF-α.

## Discussion

In this study, we established a mouse model of LPS-induced ALI and transplanted BMSCs overexpressing FoxM1 into mice through tail vein injection. With the detected track of BMSCs-FoxM1, we observed a low residency rate and short duration of residency in the lung. No expression of SPC was detected in SPC^−/−^ mice with BMSCs-FoxM1 or BMSCs-Vector transplant, however BMSCs overexpressing FoxM1 increased SPC levels relative to LPS and BMSCs-Vector groups in WT mice. We speculated that overexpression of FoxM1 enhanced the therapeutic effect of BMSCs on ARDS, possibly through a paracrine mechanism rather than by promoting differentiation of BMSCs into AT II cells in vivo. Therefore, we designed cell-level experiments to verify the paracrine effect. Our findings suggest that co-culture with BMSCs-FoxM1 protected against LPS-induced EC through the Wnt/β-catenin pathway, including anti-apoptosis and immunomodulatory effects, as well as promotion of proliferation, tube formation, and maintenance of permeability.

A surfactant is a mixture of lipid and protein synthesized and secreted by AT II cells, which reduces alveolar surface tension and prevents alveolar collapse at end-expiration. There are four pulmonary surfactant proteins: hydrophilic surfactant proteins A and D, hydrophobic surfactant protein B (SPB), and SPC [34]. SPC, a specific marker of AT II cells, is stored in lamellar bodies and secreted together with SPB and lipids. SPC accelerates the diffusion of phospholipids at the alveolar air–liquid interface and participates in pulmonary host defense. Therefore, SPC plays an important role in maintaining pulmonary homeostasis [35]. Unlike SPB-deficient mice, which are prone to progressive and fatal lung disease [36], mice with knockout of the *Sftpc* gene, which encodes SPC protein, are viable and have normal phenotypes [37]. Therefore, we examined whether BMSCs overexpressing FoxM1 differentiate into AT II cells in vivo by detecting expression of SPC in SPC-deficient mice. SPC-deficient mice exhibited no abnormal death. However, unfortunately, no expression of SPC was detected in SPC-deficient mice with implantation of BMSCs overexpressing FoxM1 or the vector only, indicating that FoxM1 overexpression was unable to facilitate differentiation of BMSCs into AT II cells in vivo.

There are various controversies surrounding whether MSCs differentiate into alveolar epithelial cells. Studies have demonstrated high pulmonary homing and epithelial differentiation efficiency of genetically modified MSCs [14], which increased interest in differentiation-inducing therapies of MSCs for ARDS. Our previous study showed that FoxM1 facilitates the differentiation of BMSCs into AT II cells by directly targeting β-catenin and activating the Wnt/β-catenin pathway in vitro [15]. However, studies have shown that MSCs exert a therapeutic paracrine effect on epithelial repair by secreting exosomes [38] or soluble factors such as keratinocyte growth factor [39], hepatocyte growth factor [40], fibroblast growth factor, and angiopoietin-1 [41]. In this study, we tracked BMSCs by bioluminescence imaging and observed that they had only a short residence in the lungs. However, we confirmed that BMSCs overexpressing FoxM1 protected against ALI by attenuating pulmonary edema and fibrosis, reducing levels of MDA and inflammatory factors (such as IL-1β, IL-6, IL-8, MIP-1α, and TGF-β), and increasing the level of SOD, indicating that the therapeutic effects of FoxM1-overexpressing BMSCs might be attributed to paracrine mechanisms. Therefore, we further designed cell-level experiments to verify the paracrine effect.

FoxM1, a member of the Fork head box (Fox) transcription factor family, is an important protein for lung development. Conditional deletion of FoxM1 in respiratory epithelium inhibits expression of surfactant proteins and lung maturation, contributing to neonatal respiratory failure [42]. The role of FoxM1 in pulmonary inflammatory diseases is well documented [29, 43–45]. In addition, expression of FoxM1 is crucial for proliferation and differentiation of AT II cells into AT I cells after ALI [46]. Xia et al. [47] revealed that FoxM1 is highly expressed in patients with bronchopulmonary dysplasia and neonatal mice with hyperoxia exposure, and selective deletion of FoxM1 in myeloid cells aggravates lung damage and inhibits alveologenesis. Thus, FoxM1 may play an important role in alveolar repair. In our study, we found that BMSCs overexpressing FoxM1 alleviated LPS-induced ALI by mitigating pulmonary pathologies, fibrosis, oxidative damage, and inflammatory responses, possibly due to the delivery of FoxM1 protein to lung tissue through a paracrine mechanism. Interestingly, FoxM1 is also associated with endothelial damage in ALI. Adherens junctions, which are mainly composed of catenins (α-, β-, and p120-catenin) and cadherin, mediate adhesion between ECs and are essential for stability of the endothelial barrier [48]. FoxM1 enhances endothelial proliferation and adherens junctions by regulating the transcription of β-catenin [45]. Zhao et al. [43] confirmed that FoxM1 expression in ECs is indispensable for bone marrow progenitor cell-induced vascular repair, as indicated by the protective effects of bone marrow progenitor cells on LPS-induced ALI are abrogated in mice with EC-restricted disruption of FoxM1. Furthermore, FoxM1 is crucial for pulmonary vascular repair mediated by hypoxia-inducible factor-1α, while liposomal delivery of FoxM1 reverses persistent lung injury and inflammation in mice with hypoxia-inducible factor-1α disruption [44].

The Wnt/β-catenin signaling pathway plays important roles in cell growth, proliferation, inflammation, fibrosis, and other pathological processes [49–51]. Our previous research found that activation of Wnt/β-catenin signaling promoted MSC-mediated protection of ECs from LPS-induced cell injury [18]. Kang et al. found that activation of Wnt/β-catenin reduced ALI-induced lung inflammation in a mouse ALI model [52]. A similar result was also observed in a study by Villar et al. [53]. Collectively, these studies demonstrate that Wnt/β-catenin signaling activation is a powerful target for the treatment of ALI/ARDS. Moreover, in our study, we found that BMSCs-FoxM1 had anti-apoptotic, proliferative, tubulogenic, and anti-inflammatory effects on LPS-induced ECs via upregulation of the Wnt/β-catenin pathway. Furthermore, our results revealed that XAV-939, a specific inhibitor of the Wnt/β-catenin pathway, partially reversed the protective effects of BMSCs-FoxM1 on LPS-induced endothelial barrier disorder, including reductions of Wnt/β-catenin, VE-cadherin, and BCL-2 levels; increased BAX levels, and inhibition of anti-apoptosis. These results indicate that BMSCs-FoxM1 protected against LPS-induced ALI/ARDS partially through activation of the Wnt/β-Catenin signaling pathway. Taken together, these findings may facilitate the development of new MSC-based therapeutic approaches to treat ALI/ARDS.

## Conclusion

Collectively, our study indicated that FoxM1 overexpression enhanced the therapeutic effect of BMSCs on ARDS, possibly through a paracrine mechanism rather than by promoting the differentiation of BMSCs into AT II cells in vivo. In addition, FoxM1 overexpression prevented LPS-induced EC barrier dysfunction partially through activation of the Wnt/β-catenin signaling pathway in vitro. These findings suggest that FoxM1 gene-modified BMSCs may be an attractive strategy for the treatment of ALI/ARDS**.**

## Supplementary Information

**Additional file 1**. Original uncropped blots of the text WB figure in the manuscript.

## Data Availability

The data that support the findings of this study are available from the corresponding author upon reasonable request.

## Abbreviations

ALI: Acute lung injury
ARDS: Acute respiratory distress syndrome
FoxM1: Fork head box protein M1
Fox: Fork head box
MSCs: Mesenchymal stem cells
BMSCs: Bone marrow-derived MSCs
SD: Sprague–Dawley
AT II: Alveolar type II
AT I: Alveolar type I
SPC^−^^/^^−^: SPC gene knockout
ECs: Endothelial cells
H&E: Hematoxylin and eosin
LPS: Lipopolysaccharide
IL-1β: Interleukin 1beta
IL-6: Interleukin 6
IL-8: Interleukin 8
MIP-1α: Macrophage inflammatory protein 1 alpha
IL-4: Interleukin 4
IL-10: Interleukin 10
TNF-α: Tumor necrosis factor alpha
TGF-β: Transforming growth factor beta
BALF: Bronchoalveolar lavage fluid
IOD: Integral optical density
MDA: Malondialdehyde
GSH: Glutathione
SOD: Superoxide dismutase
BLI: Bioluminescence imaging
BCA: Bicinchoninic acid
D: Dry weight
W: Wet weight
WT: Wildtype
SP-B: Surfactant protein B
SP-C: Surfactant protein C
EdU: 5-Ethynyl-20-deoxyuridine
AV-FITC/PI: Annexin V-fluorescein isothiocyanate/propidium iodide
MOI: Multiplicity of infection
ELISA: Enzyme linked immunosorbent assay
qRT-PCR: Quantitative real-time polymerase chain reaction
SEM: Standard error of the mean
