# Supplementary figures and images for: Overexpression of FoxM1 optimizes the therapeutic effect of bone marrow mesenchymal stem cells on acute respiratory distress syndrome
Source: Stem Cell Res Ther. 2023 Feb 14;14:27. doi: 10.1186/s13287-023-03240-8 (PMC9926819; doi:10.1186/s13287-023-03240-8)

Fig.1

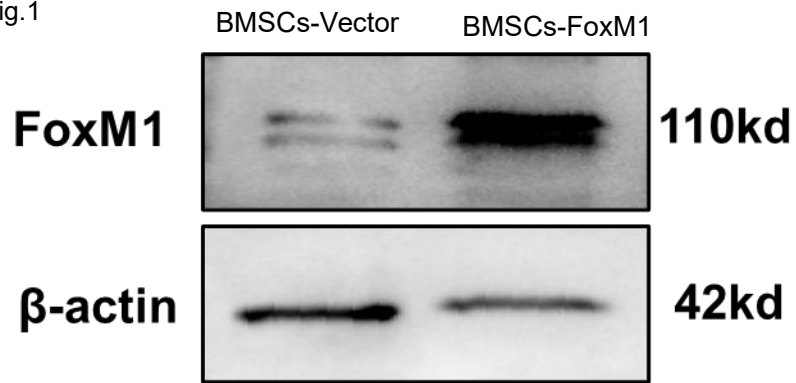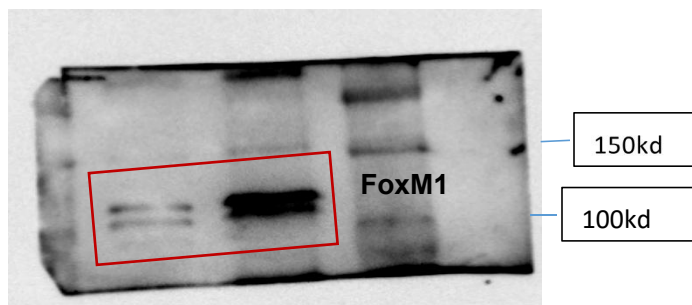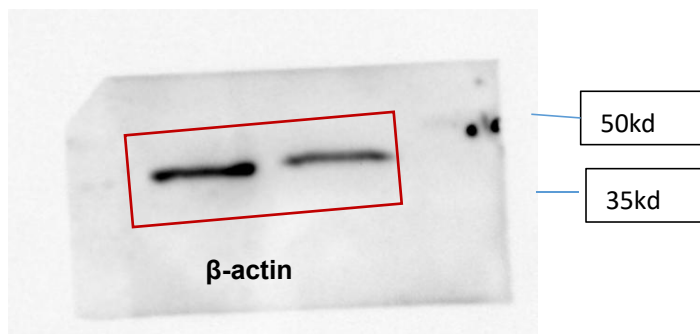

Fig. 7

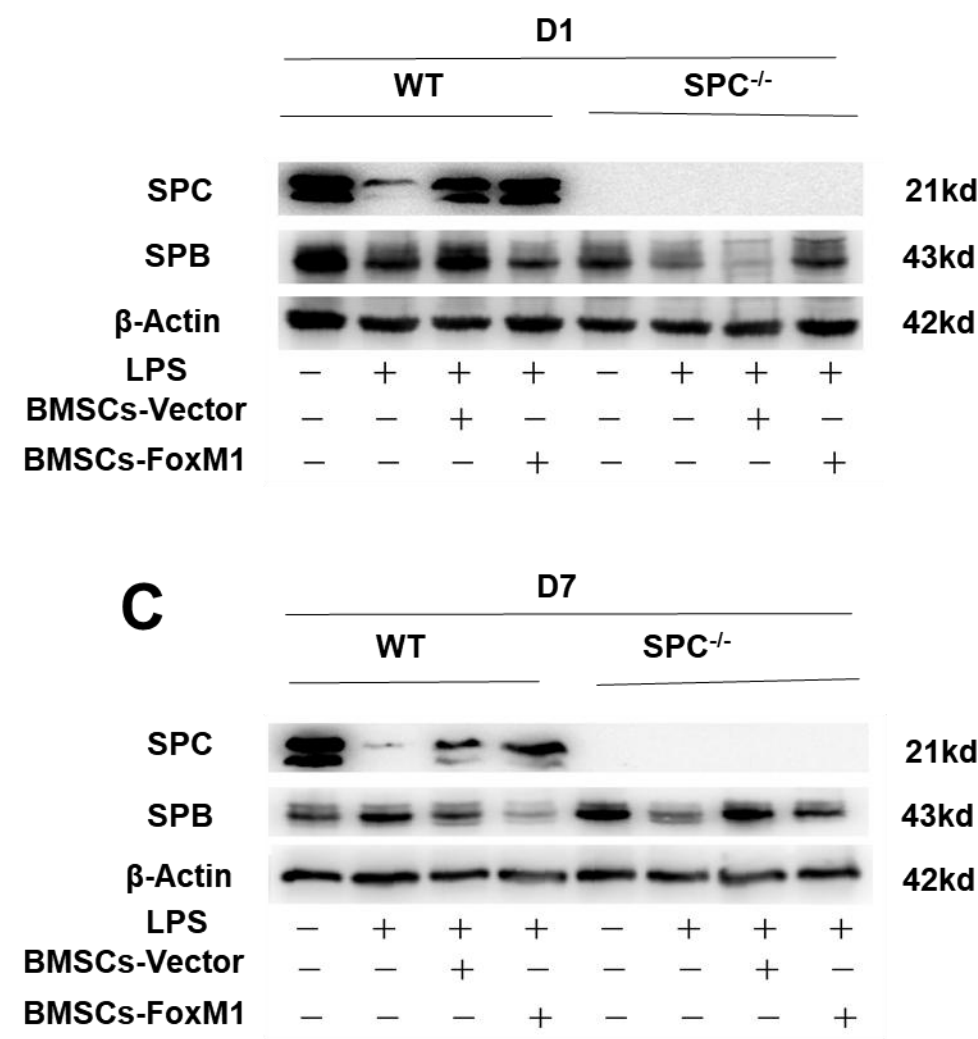

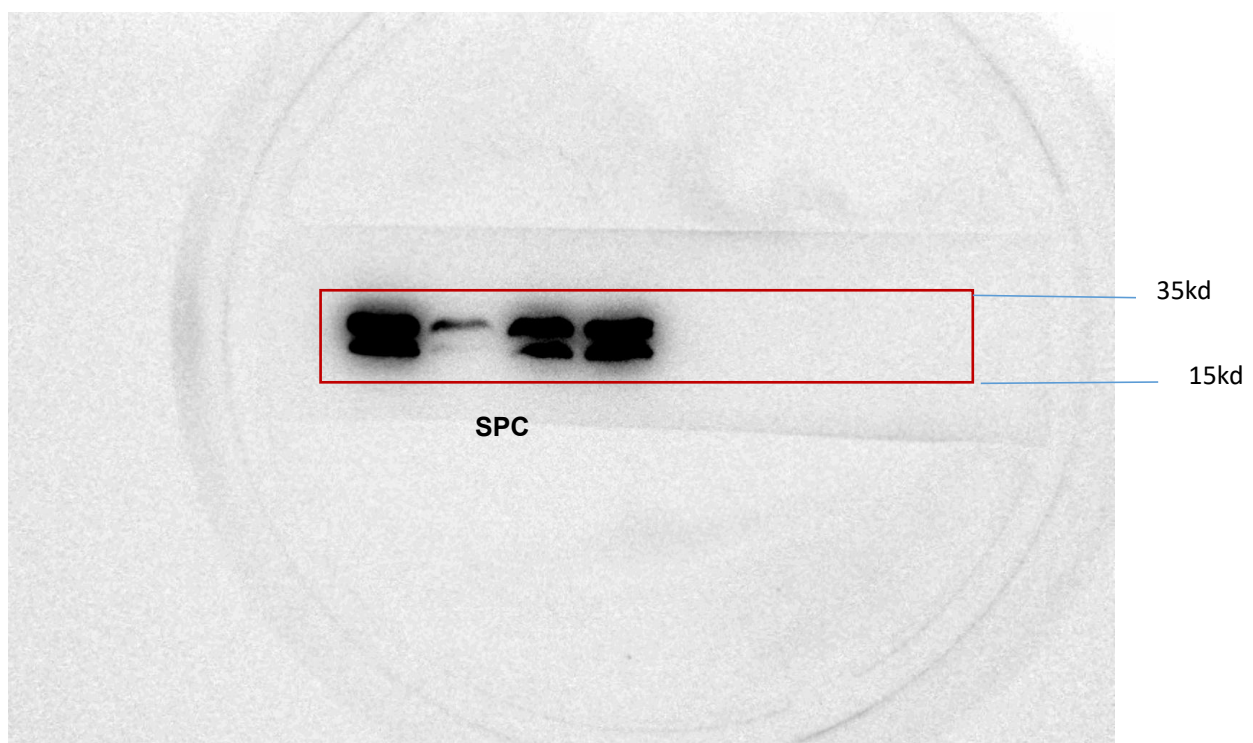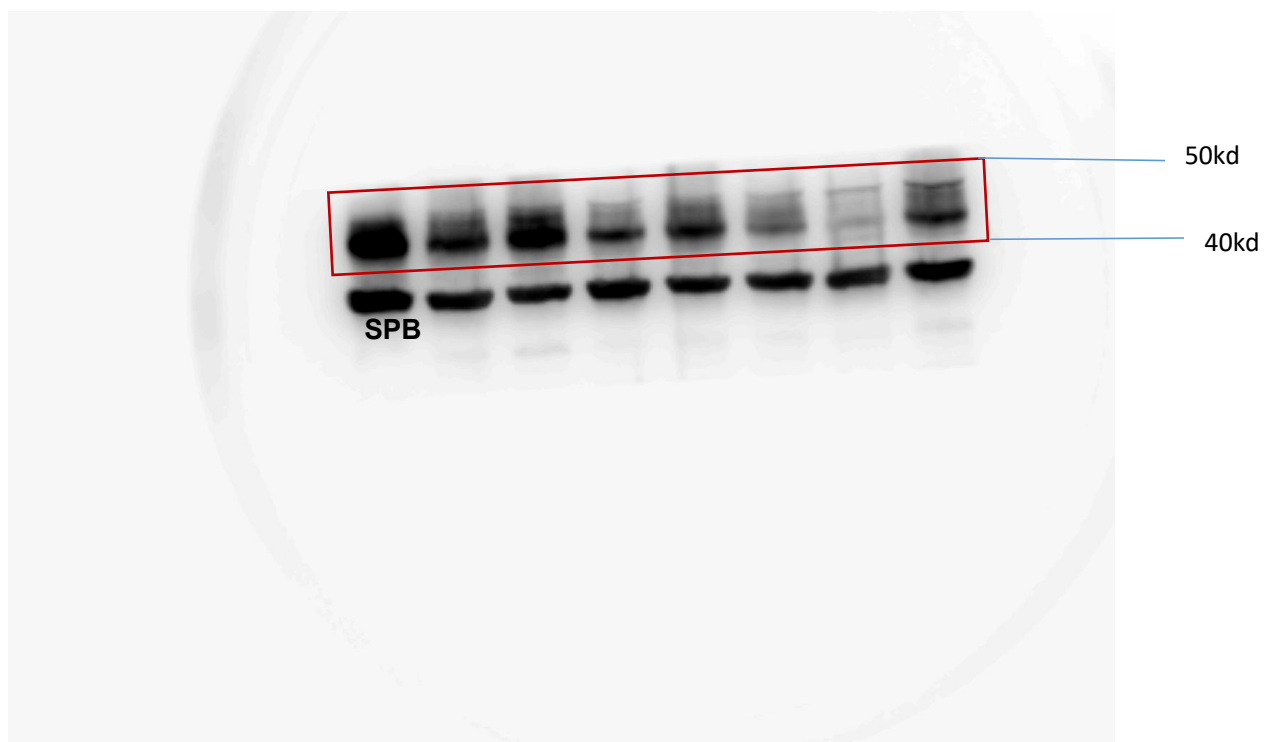

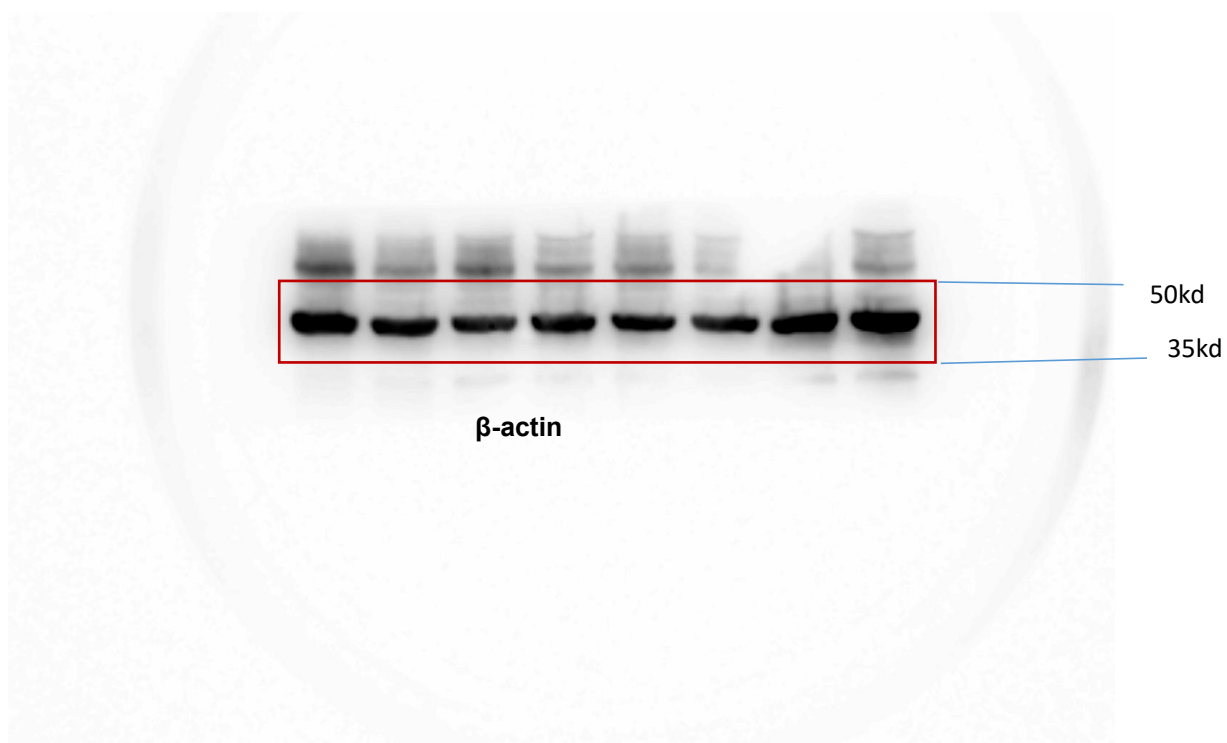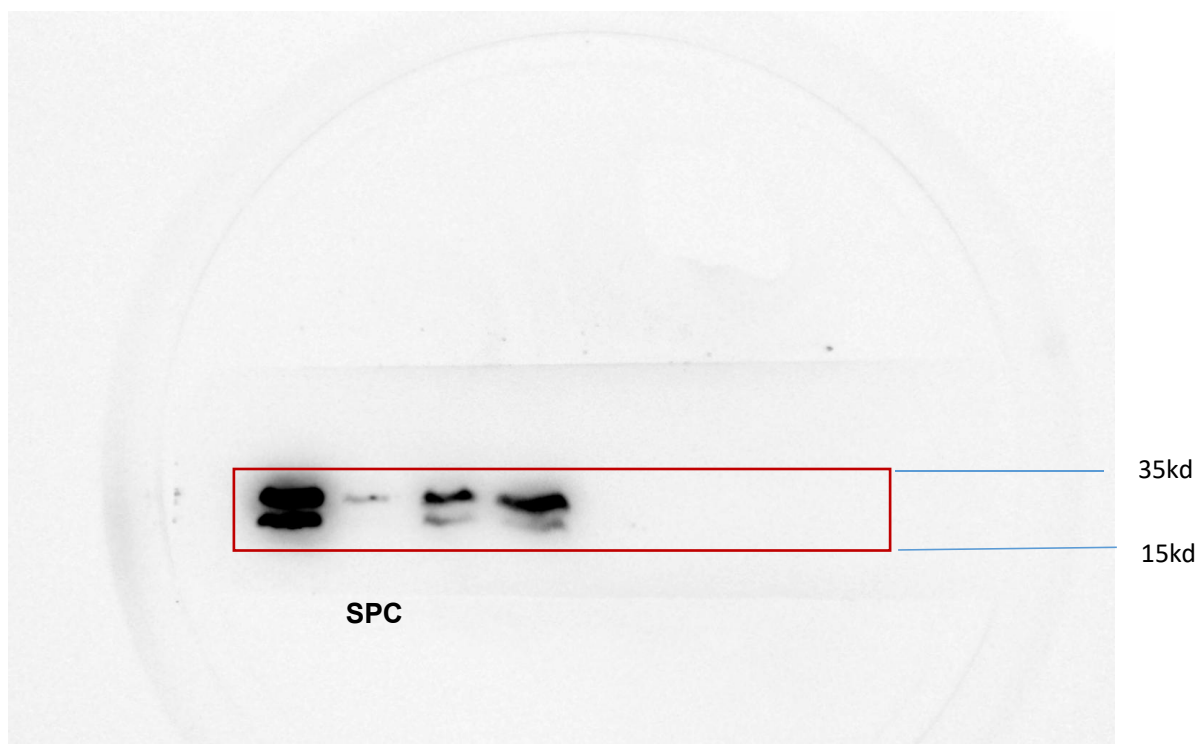

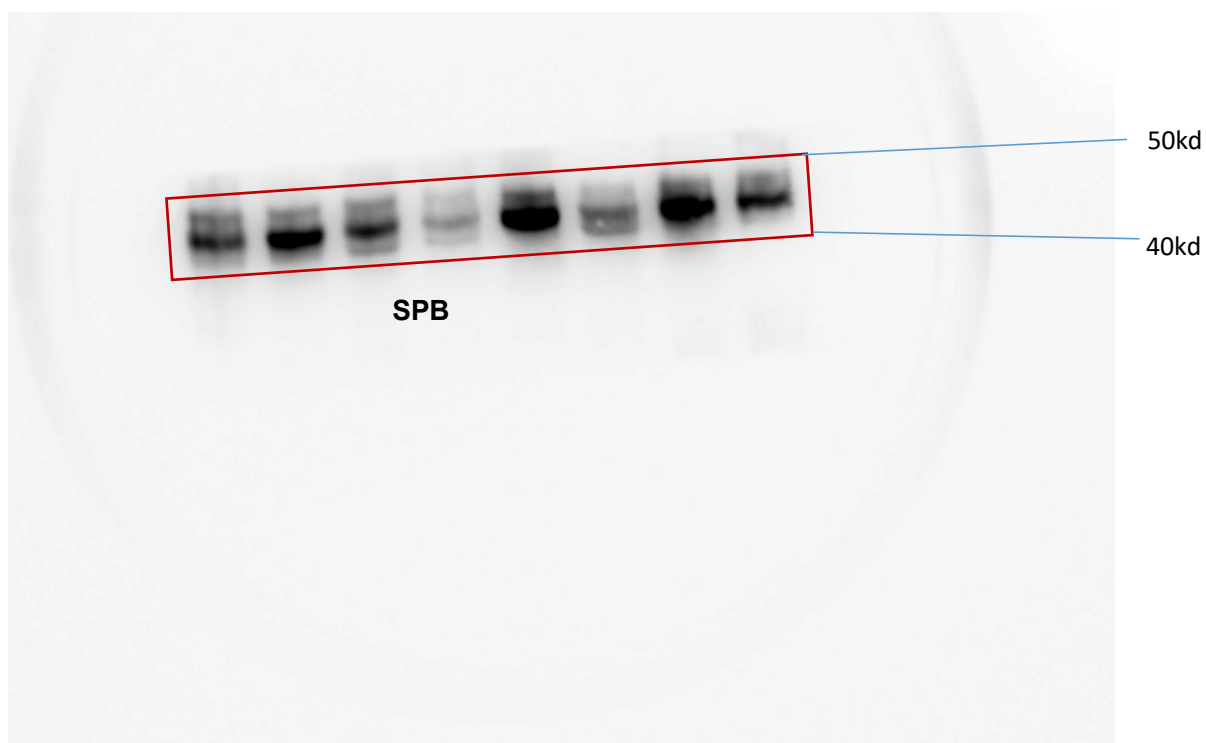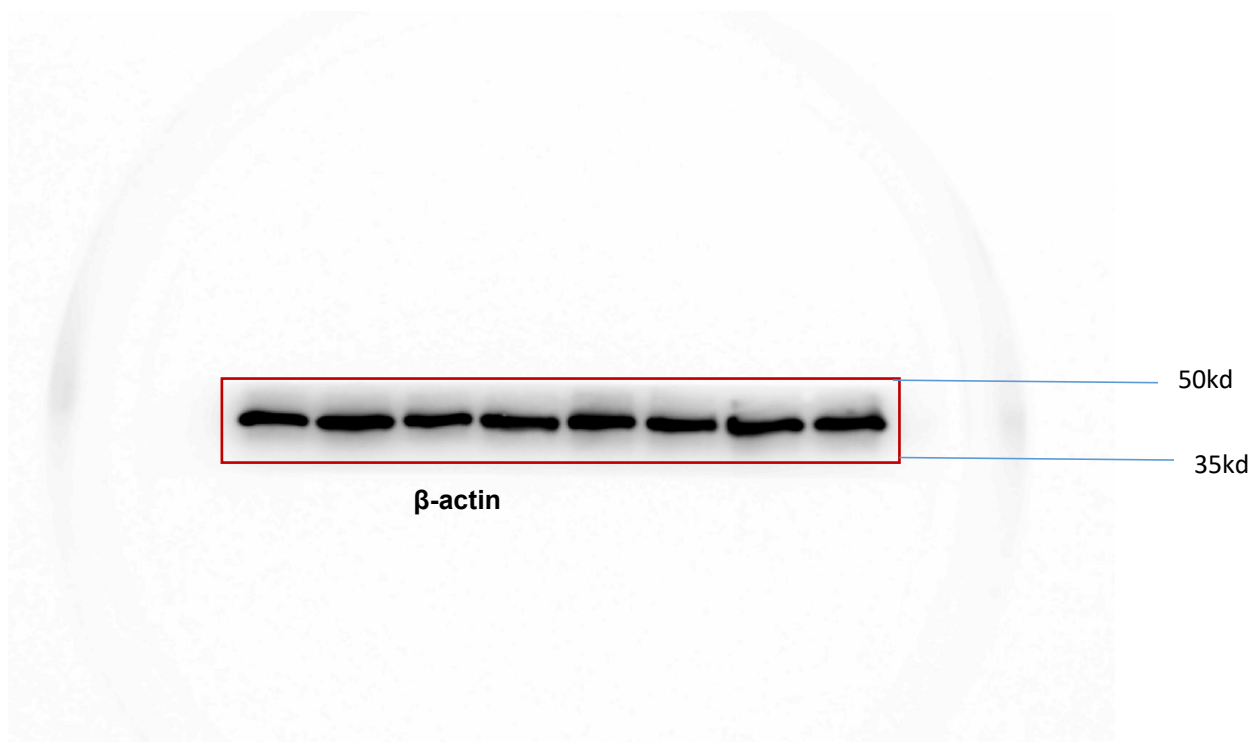

Fig.10

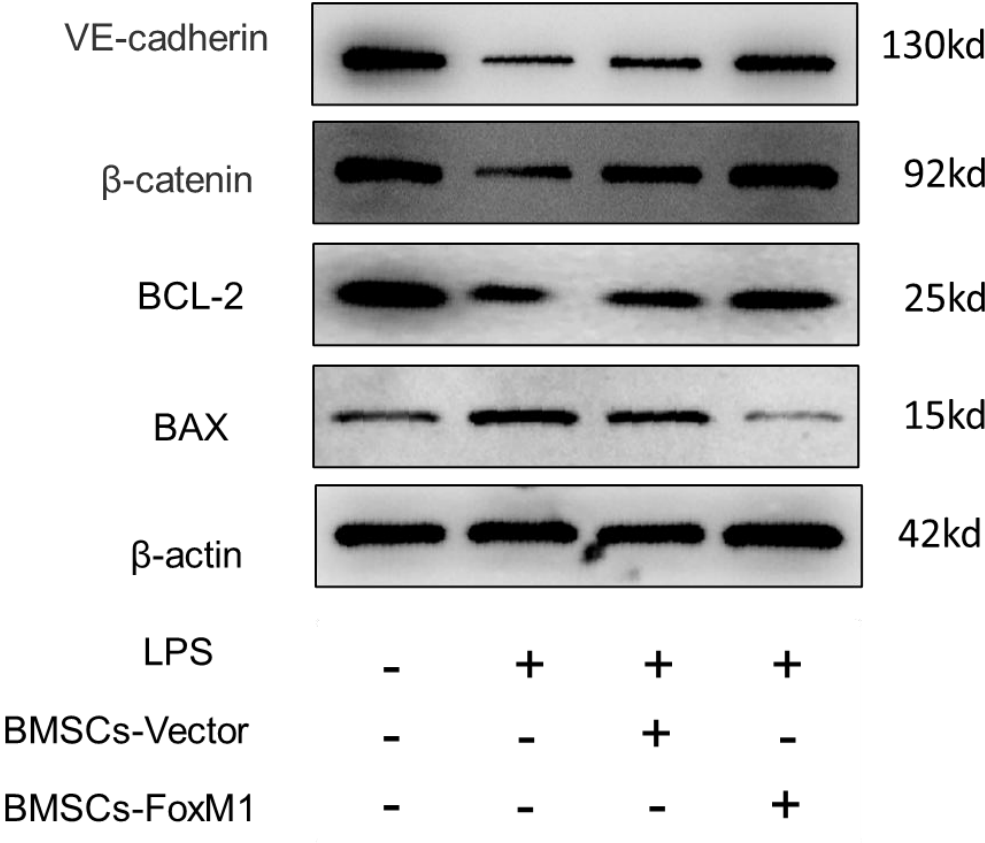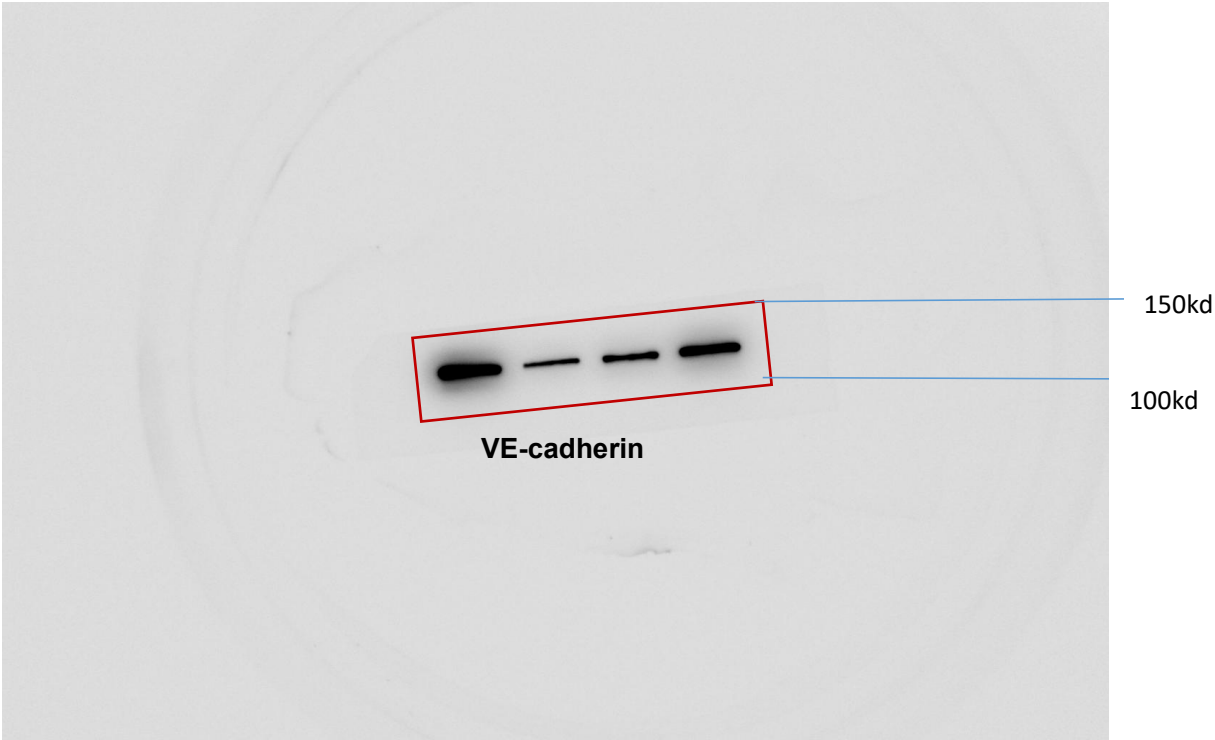

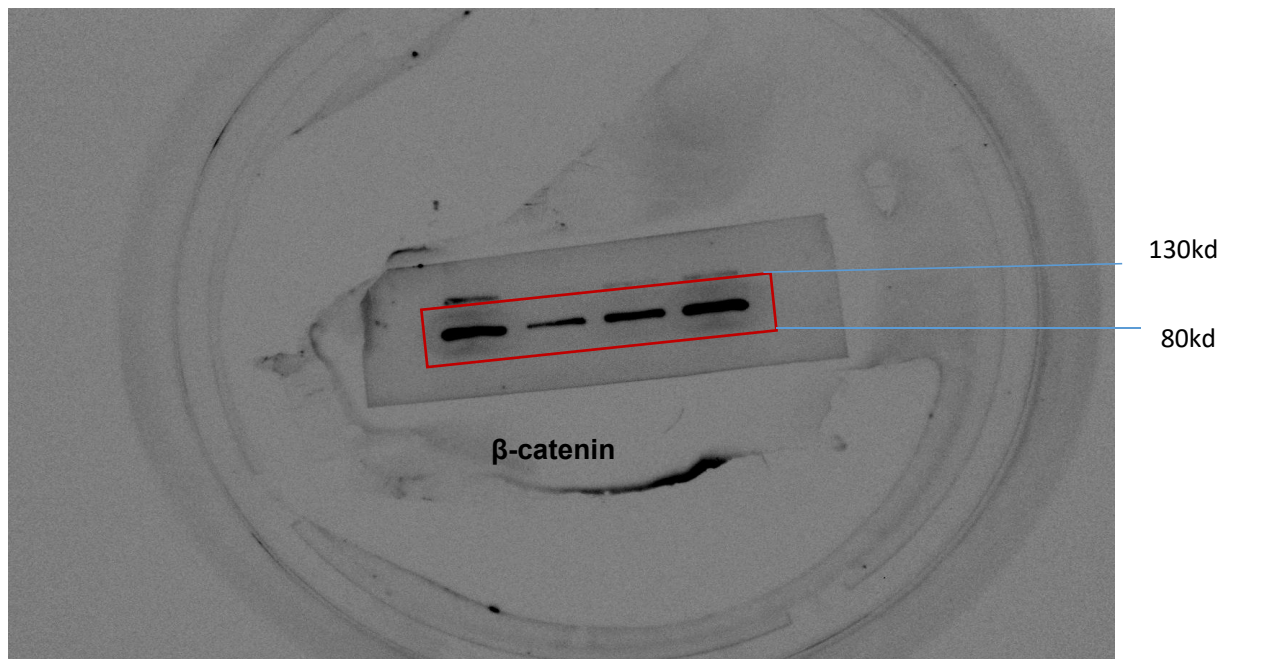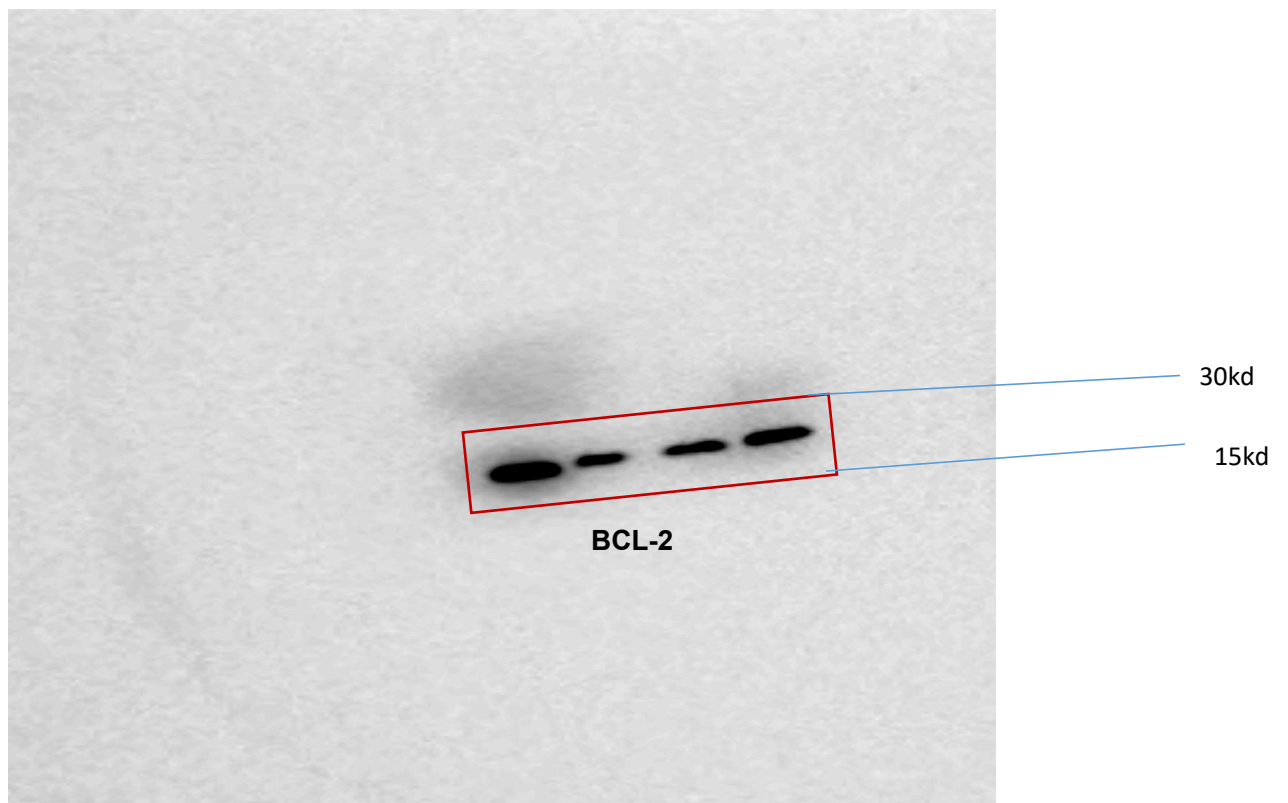

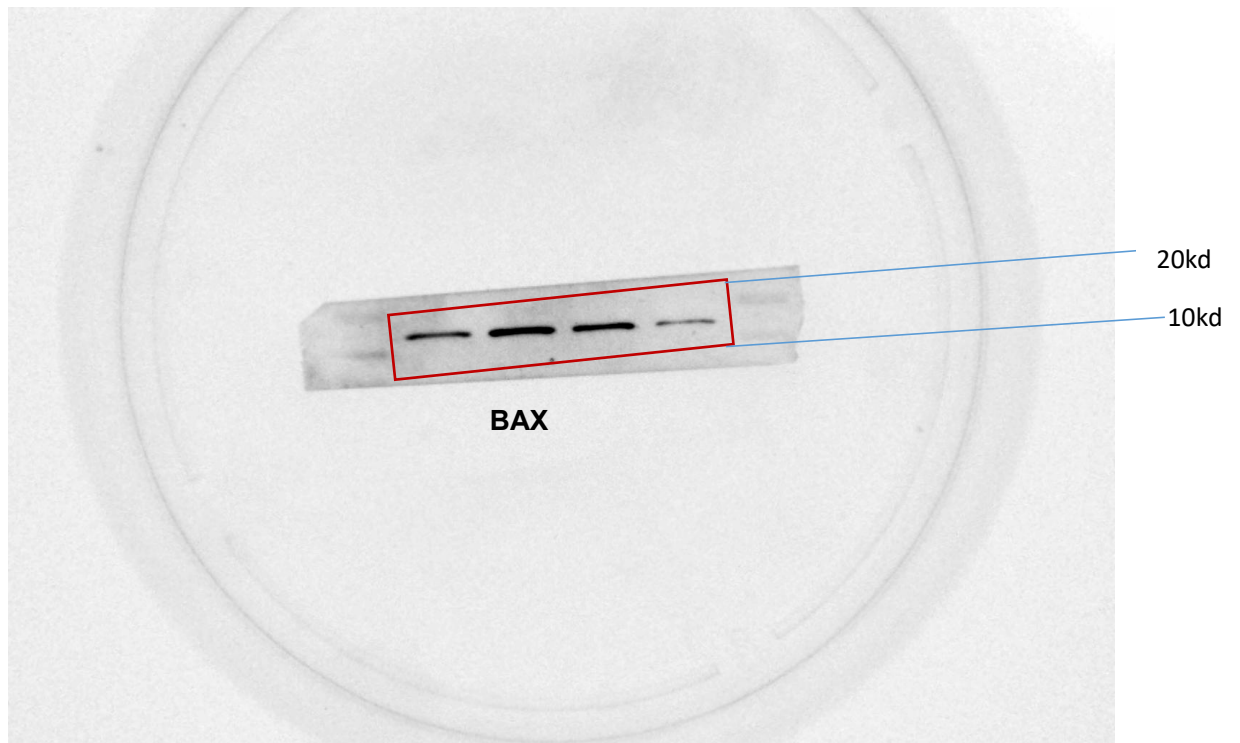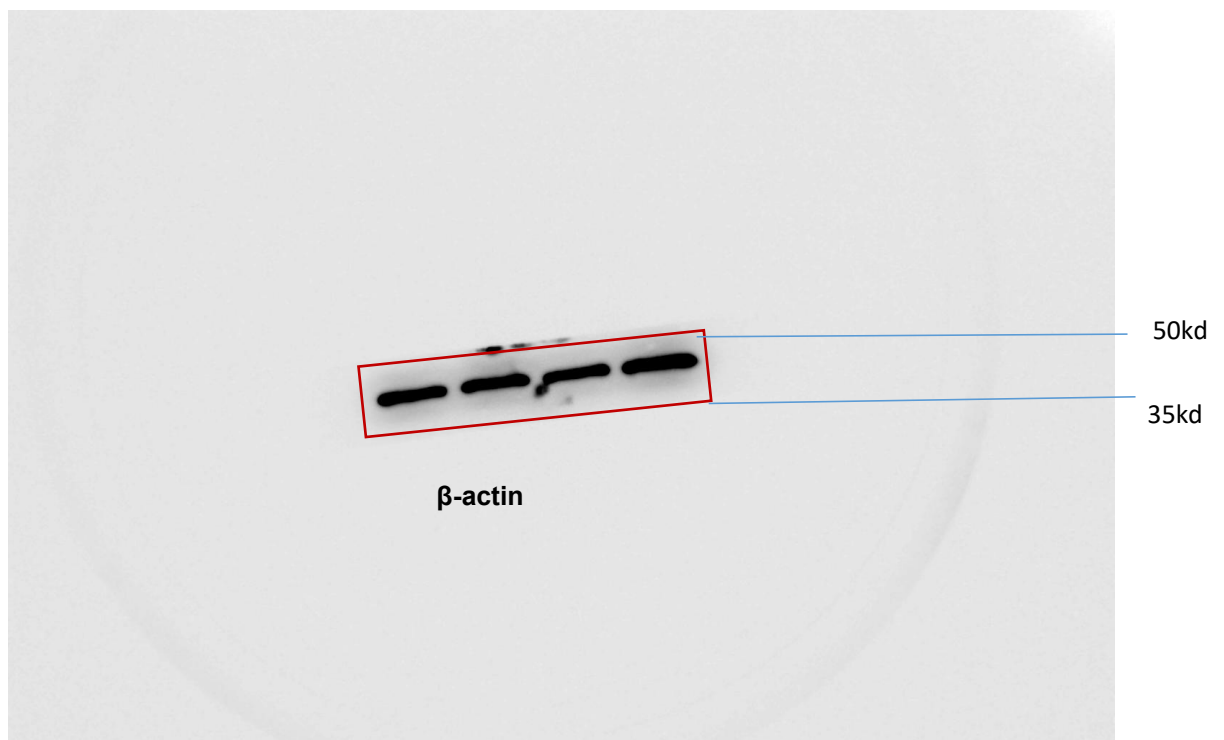

Fig. 11.

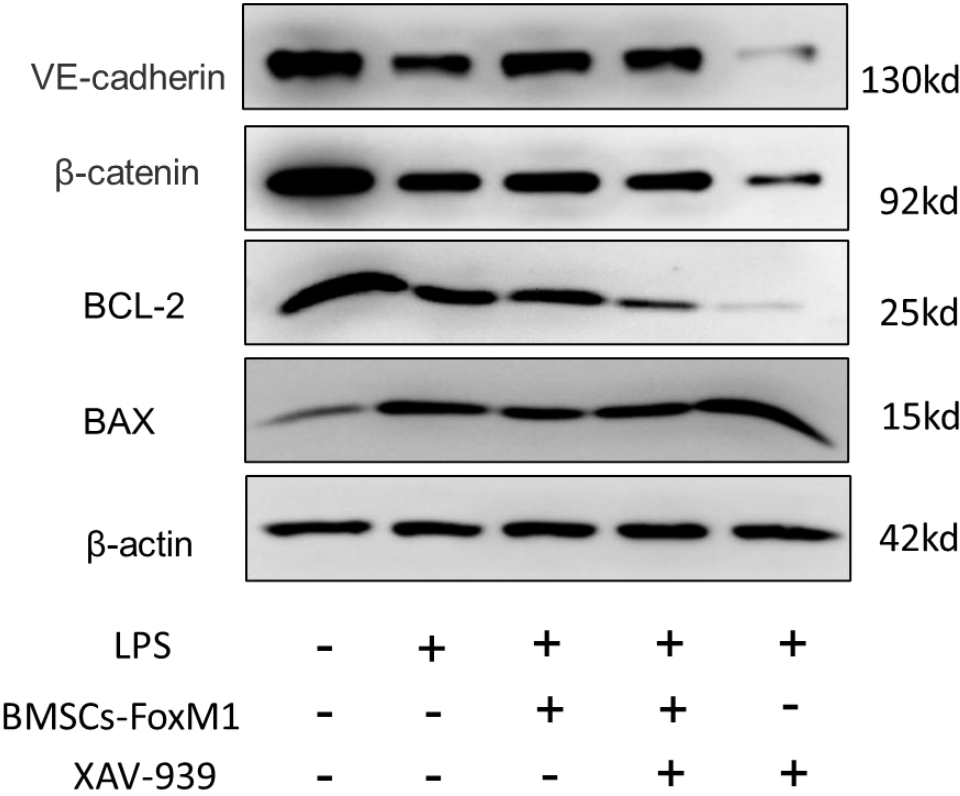

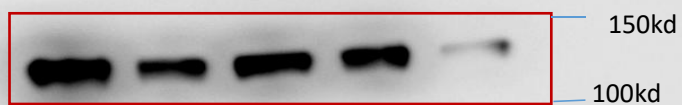

**VE-cadherin**

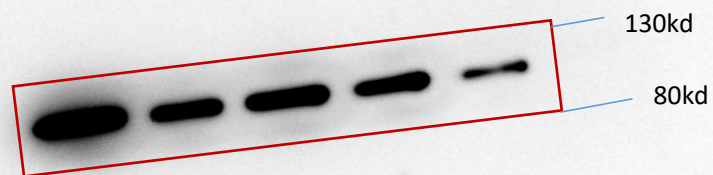

β-catenin

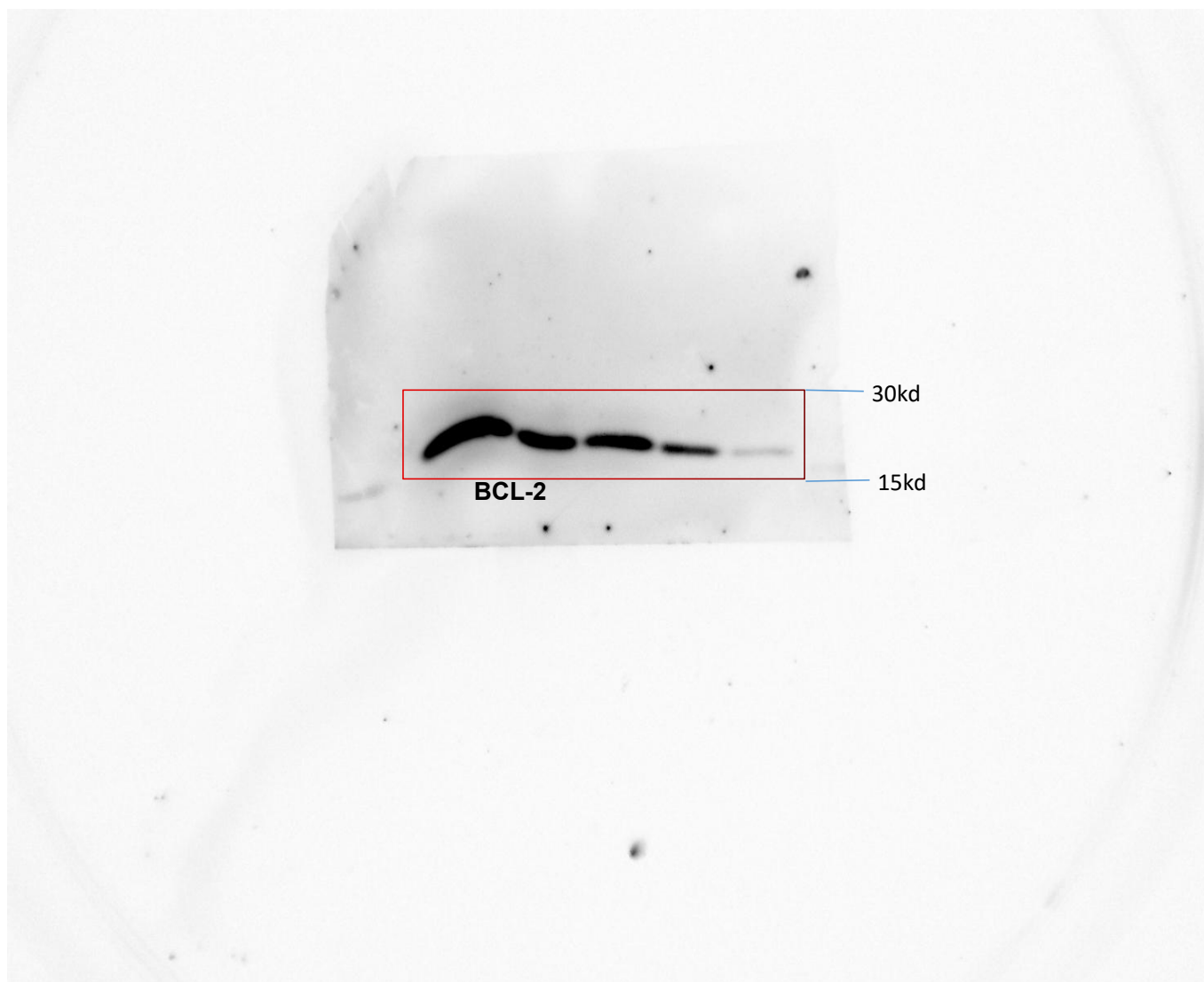

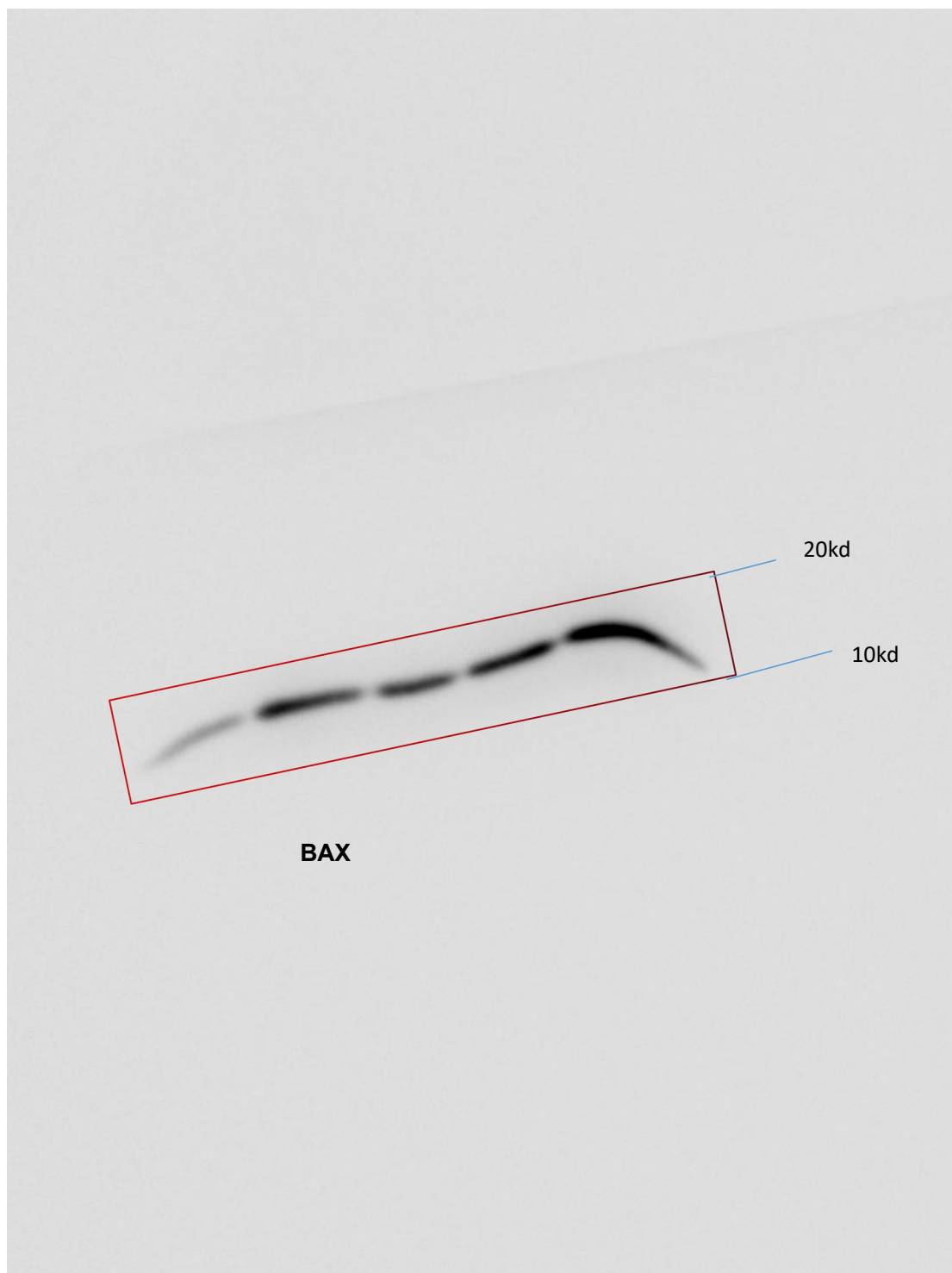

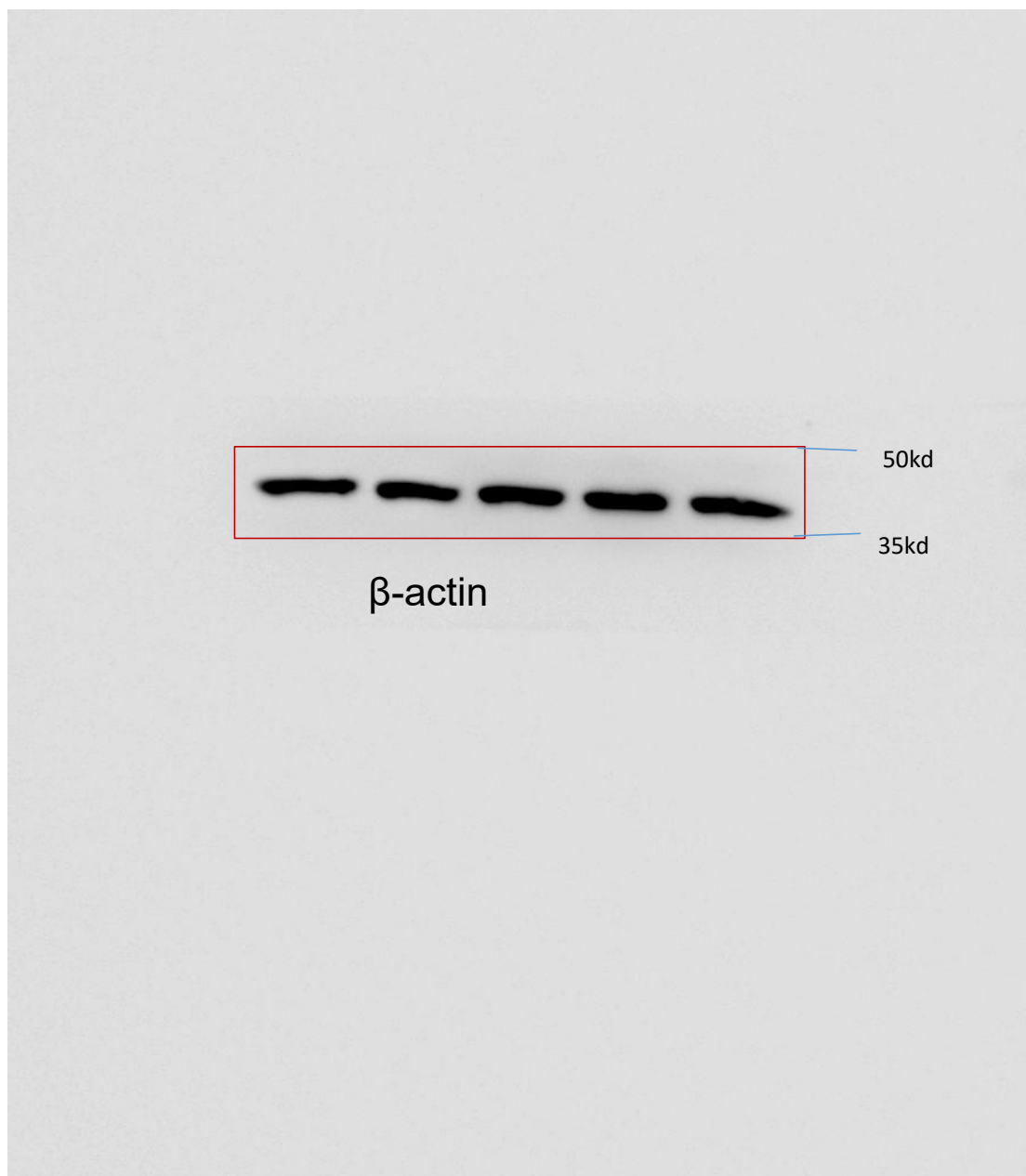

Supplement: Supplementary file 1 — Additional file 1. Original uncropped blots of the text WB figure in the manuscript. [file 13287_2023_3240_MOESM1_ESM.pdf]
